# Supplementary material for: Human iPSC models of myocardial infarction: From injury to repair and therapeutic discovery
Source: Cell Rep Med. 2026 Sep 25;7(10):103079. doi: 10.1016/j.xcrm.2026.103079 (PMC13710544; doi:10.1016/j.xcrm.2026.103079)
Supplement: Supplementary file 1 [file mmc1.pdf]

Cell Reports Medicine, Volume *7*

Supplemental information

Human iPSC models of myocardial infarction: From injury to repair and therapeutic discovery

Benjamin B. Johnson, Christine L. Mummery, and Richard P. Davis

Supplementary Table 1. Comparison of key strengths and limitations of hiPSC-based cardiac models and animal models in cardiovascular research

Feature	hiPSC-based cardiac models	Animal models
**Species relevance**	Human genetic background and human-relevant expression patterns	Provide intact physiology, but species differences can limit translation
**Patient specificity**	Can capture patient- or genotype-specific phenotypes	Limited patient specificity
**Ethics**	Avoid many animal-use issues, but still require donor consent and ethical oversight	Subject to animal ethics regulations and welfare constraints
**Scalability**	Expandable and compatible with medium- to high-throughput workflows	Lower throughput and constrained by cost, logistics, and animal use
**Maturity**	Often developmentally immature and may require maturation strategies	Native adult tissue context, although experimental animals may not reflect typical patient age or comorbidity
**Biological complexity**	Controlled and modular, but lack complete systemic, vascular, immune, and neurohumoral context	Capture integrated organ- and organism-level physiology
**Genetic manipulation**	Amenable to targeted engineering and isogenic study design	Possible, but generally slower and more resource-intensive
**Drug testing**	Useful for human-relevant mechanistic studies and screening workflows	Important for integrated pharmacology and in vivo efficacy, but may not predict human responses
**Disease modelling**	Particularly strong for human genetic disease and defined cellular phenotypes; increasingly useful for injury and remodelling models	Useful for some genetic and chronic diseases; strong for integrated injury, remodelling, and functional outcomes in vivo
**Cost**	Lower marginal cost per condition once established	Higher cost per experiment

Supplementary Table 2: hiPSC models of MI and IHD: model format, cell composition, injury method, and disease phase modelled

#	Paper title	Model format	Cell composition	Injury method	Disease phase(s) modelled	Ref
2D systems						
1	hiPSC-based platform for modelling cardiac ischemia	2D monolayer	hiPSC-CMs only	Hypoxia + metabolic stress followed by reoxygenation (low O_2, altered medium composition)	Acute I/R injury and CM death. Models mitochondrial dysfunction, Ca^{2+} handling changes, and CM viability	72
2	Modelling ischemia-reperfusion injury (IRI) in vitro using metabolically matured induced pluripotent stem cell-derived cardiomyocytes	2D monolayer with metabolic maturation	hiPSC-CMs only; matured in galactose- and fatty acid-containing medium	Ischaemia: 0% O_2 for 2h in glucose-free medium at pH 6.2 or 7.4. Reperfusion: 20% O_2 with glucose for 4h	Acute I/R injury and CM death. Provides well-defined CM death and mitochondrial injury endpoints	73
3	In vitro model of ischemic heart failure using human induced pluripotent stem cell-derived cardiomyocytes	2D monolayer with chemical ischaemia	Primarily hiPSC-CMs; some variants include CFs or ECs	Chemical ischaemia using metabolic inhibitors, with induction of CM arrhythmic phenotypes	Acute ischaemic-like metabolic stress and CM death. Focuses on ATP depletion, ROS and CM viability; not designed to follow later repair or remodelling	74
3D systems						
4	Bioreactor-based 3D human myocardial ischemia/reperfusion in vitro model: a novel tool to unveil key paracrine factors upon acute myocardial infarction	3D aggregates in a bioreactor	hiPSC-CMs	Controlled perfusion hypoxia (<0.4% O_2) with low glucose/nutrient-limited medium for 5h, followed by normoxic reperfusion	Acute I/R injury and early CM responses, including apoptosis, necrosis, sarcomere disorganisation, and mitochondrial damage	82
5	In vitro aged, hiPSC-origin engineered heart tissue models with age-dependent functional deterioration to study myocardial infarction	3D in hydrogel	hiPSC-CMs; young versus aged by culture duration	Oxidative H_2O_2 insult or 1% O_2 for 48h, followed by reoxygenation	Acute I/R-like injury with age-dependent susceptibility, ECM stiffening, better recovery in younger tissues, and reduced survival in aged tissues	83
6	Modelling acute myocardial infarction and cardiac fibrosis using human induced pluripotent stem cell-derived multi-cellular heart organoids	3D cardiac organoid	hiPSC-derived organoids containing CMs, CF-like stromal cells, ECs, SMC-/ epicardial-like cells	$CoCl_2$ and glucose depletion followed by 72h high glucose, high Ca^{2+}; fibrosis progression induced with TGF-β1	Acute MI-like injury and high glucose reperfusion, followed by organoid remodelling, CM death, inflammatory/ fibrotic transcriptional responses in fibroblast-like cells, ECM deposition, and neovascular changes	90

7	Human Tissue-Engineered Model of Myocardial Ischemia-Reperfusion Injury	Engineered heart tissue	hiPSC-CMs in collagen I and fibrinogen hydrogel	Nutrient deprivation and 0% O_2 for 6h, followed by normoxia, high glucose and Ca^{2+} for 3h	Acute I/R injury and high glucose reperfusion, with CM disarray and ROS accumulation	81
8	Biofabrication of advanced in vitro 3D models to study ischaemic and doxorubicin-induced myocardial damage	3D cardiac spheroids	hiPSC-CMs, primary human CFs and primary human coronary artery endothelial cells	0% O_2 for 20h followed by reperfusion at 5% O_2 for 17h	Acute I/R injury with CM death, reduced contractility and early MI-associated gene expression changes	86
9	Cardioids reveal self-organizing principles of human cardiogenesis	3D cardiac organoid (cardioid)	hiPSC-derived cardioids; self-organising “chamber-like” organoids containing CMs, ECs, CF-like cells	Focal cryoinjury applied to the cardioid	Localised necrosis and early healing/ remodelling. Captures acute CM death in a defined region followed by ECM deposition, fibronectin/collagen accumulation and partial functional recovery, mimicking aspects of infarction and early wound healing	65
10	Human cardiac organoids for the modelling of myocardial infarction and drug cardiotoxicity	3D cardiac spheroids	hiPSC-derived cardiac organoids containing CMs, ECs, and fibroblasts/stromal cells; self-organising dense structure (~150 µm radius)	Spontaneous hypoxic core due to O_2 diffusion limits in dense organoids cultured at 10% O_2 with 1 µM norepinephrine stimulation for 10 days to mimic infarct-like stress	Acute hypoxic injury and metabolic stress response. Captures a diffusion-limited hypoxic core (mimicking subendocardial ischaemia), ROS accumulation, cell death, and hypertrophy	87
11	Engineered model of heart tissue repair for exploring fibrotic processes and therapeutic interventions	Engineered heart tissue	hiPSC-CMs and -CFs in hydrogel	Cryoinjury	Acute damage followed by fibroblast migration, wound healing responses, and fibrotic remodelling	84
Bioengineered devices						
12	Endothelial extracellular vesicles contain protective proteins and rescue ischemia-reperfusion injury in a human heart-on-chip	Laminar cardiac tissues on film cantilevers	hiPSC-CMs	1% O_2 hypoxia with glucose deprivation for 3h, followed by 1.5h normoxia with glucose for reperfusion	Acute I/R injury and CM injury/death, followed by assessment of cardioprotective EC-derived extracellular vesicles	91
13	Biowire Model of Interstitial and Focal Cardiac Fibrosis	Aligned cardiac tissues on conductive wires	hiPSC-CMs and primary ventricular CFs	High CF:CM ratio to model interstitial and focal fibrosis; no hypoxia or I/R injury	Late-stage fibrotic remodelling with increased tissue stiffness, impaired contractility and calcium handling, and pro-arrhythmic features	94

14	Human cardiac fibrosis-on-a-chip model recapitulates disease hallmarks and can serve as a platform for drug testing	3D cardiac tissues anchored to flexible PDMS rods	hiPSC-CMs and primary human CFs	TGF-β1-induced fibrosis; no hypoxia or I/R injury	Fibrotic remodelling with reduced contractile function, collagen deposition and increased tissue stiffness, followed by anti-fibrotic drug testing	93
Heart-on-chip systems						
15	Human Heart Anoxia and Reperfusion Tissue (HEART) model for the rapid study of exosome bound miRNA expression as biomarkers for myocardial infarction	3D cardiac hydrogel with EC-lined vascular channel	hiPSC-CMs in human heart ECM hydrogel and hiPSC-ECs in vascular channel	Flow cessation and 0.1% O_2 for 3h, followed by restoration of normoxia and perfusion for 3h	Models acute MI-like flow restriction and reperfusion, with severe hypoxia during flow cessation followed by restored oxygenation/perfusion; captures early CM death and MI-associated gene expression changes	96
16	Cardiac ischemia on-a-chip to investigate cellular and molecular response of myocardial tissue under hypoxia	Aligned 3D cardiac tissues in a microfluidic chip with embedded microposts	hiPSC-CMs and primary human CFs	1% O_2 hypoxia for 24h, followed by reoxygenation for 24h	Acute I/R-like injury with CM loss; captures transcriptomic changes, altered contractility, and fibrotic response	97
17	Immune-integrated cardiac fibrosis-on-a-chip: a 3D microfluidic device for region-specific immune-cardiac crosstalk in the fibrotic heart	Region-patterned 3D cardiac tissue with valve-controlled cell delivery	hiPSC-CMs and -CFs; hiPSC-macrophages added region-specifically after patterning	No hypoxia or I/R injury; TGF-β treatment and spatial patterning creates scar, border-zone, and healthy-like regions	Post-MI fibrotic remodelling and immune–fibrosis crosstalk, with region-specific macrophage effects; does not model ischaemia or acute CM death	98
