# Supplementary material for: Human iPSC models of myocardial infarction: From injury to repair and therapeutic discovery
Source: Cell Rep Med. 2026 Sep 25;7(10):103079. doi: 10.1016/j.xcrm.2026.103079 (PMC13710544; doi:10.1016/j.xcrm.2026.103079)
Supplement: Supplementary file 2 [file mmc2.pdf]

Cell Reports Medicine

CellPress
OPEN ACCESS

Review

Human iPSC models of myocardial infarction: From injury to repair and therapeutic discovery

Benjamin B. Johnson,[1,2,*] Christine L. Mummery,[1,2,*] and Richard P. Davis[1,2,*]
[1]Department of Anatomy and Embryology, Leiden University Medical Center, 2300RCLeiden, the Netherlands
[2]The Novo Nordisk Foundation Center for Stem Cell Medicine, reNEW, Leiden University Medical Center, 2300RC Leiden, the Netherlands
*Correspondence: b.b.johnson@lumc.nl (B.B.J.), c.l.mummery@lumc.nl (C.L.M.), r.p.davis@lumc.nl (R.P.D.)
https://doi.org/10.1016/j.xcrm.2026.103079

SUMMARY

Myocardial infarction (MI) remains a leading cause of death worldwide, and therapies that limit injury, promote repair, and restore cardiac function remain limited. Human induced pluripotent stem cells (hiPSCs) provide a renewable source of patient-specific cardiac cells and have enabled the development of increasingly complex *in vitro* models that incorporate cardiomyocytes together with supporting stromal and vascular cell types. These models are now being used to study multiple stages of MI pathophysiology and are increasingly being applied in therapeutic discovery and regenerative strategies. In this review, we summarize hiPSC-based cardiac models spanning 2D cultures to advanced 3D tissues, organoids, and heart-on-chip systems, evaluating how well they capture key features of human MI. We further discuss hiPSC applications in therapeutic discovery, drug screening, precision medicine, and regenerative repair. Finally, we highlight priorities for the field, including improved physiological complexity, stronger translational robustness, bioengineering strategies, and integration with multi-omics.

THE GLOBAL BURDEN OF CARDIOVASCULAR DISEASE AND UNMET THERAPEUTIC NEED

Cardiovascular disease (CVD) remains the leading cause of death worldwide, accounting for an estimated 20.5 million deaths in 2021 and projected to exceed 35 million annually by 2050 as populations grow and age. This rise reflects both population growth and aging, with increasing numbers of older adults at high risk of myocardial infarction.[1,2] The absolute burden of CVD also continues to rise, driven by persistent metabolic and lifestyle risk factors including hypertension, obesity, poor diet, and physical inactivity.[3] Healthcare expenditure already exceeds €280 billion in the European Union and $400 billion in the United States and is projected to increase markedly by mid-century, placing further strain on healthcare systems.[4,5]

Ischemic heart disease (IHD) is a major contributor to cardiovascular mortality.[1,6,7] IHD encompasses myocardial ischemic syndromes in which blood supply is insufficient to meet oxygen demand, most commonly because of atherosclerotic coronary artery disease. Acute atherosclerotic plaque rupture or erosion, followed by thrombus formation and coronary blood flow obstruction, is the most common mechanism leading to myocardial infarction (MI), defined as cardiomyocyte death caused by prolonged ischemic injury. Myocardial ischemia can also arise from non-atherosclerotic causes, including coronary embolism, vasospasm, and microvascular dysfunction, while stress-induced cardiomyopathies such as Takotsubo syndrome can mimic acute MI.[6,8–10] Additionally, outcomes after MI differ between men and women, owing in part to differences in symptom presentation, diagnostic delay, comorbidities, and treatment patterns.[6,7,11] In large infarcts, the loss of up to one billion cardiomyocytes, representing approximately 25% of the left ventricular cardiomyocyte pool, drives adverse remodeling and progression to heart failure.[9,12–14]

Rapid restoration of coronary perfusion by percutaneous coronary intervention remains the cornerstone of acute MI management and has substantially improved survival and post-infarct outcomes.[15] Reperfusion must be achieved promptly to limit irreversible necrosis and is typically followed by multidrug secondary prevention and cardioprotective strategies.[8,10,13] Nevertheless, mortality and progression to heart failure remain substantial because of the limited regenerative capacity of the adult human heart and the subsequent inflammatory injury and maladaptive remodeling after reperfusion. A substantial proportion of sudden cardiac deaths after MI are mediated by malignant ventricular arrhythmias arising from ischemia-induced and post-infarct electrical instability rather than by mechanical pump failure alone.[14,16,17] These limitations highlight the need for improved cardioprotective and regenerative therapies and more predictive human-relevant preclinical models. Developing such models requires defining the cellular and molecular processes occurring during and after ischemic injury.

© 2026 The Authors. Published by Elsevier Inc.
This is an open access article under the CC BY license (http://creativecommons.org/licenses/by/4.0/).

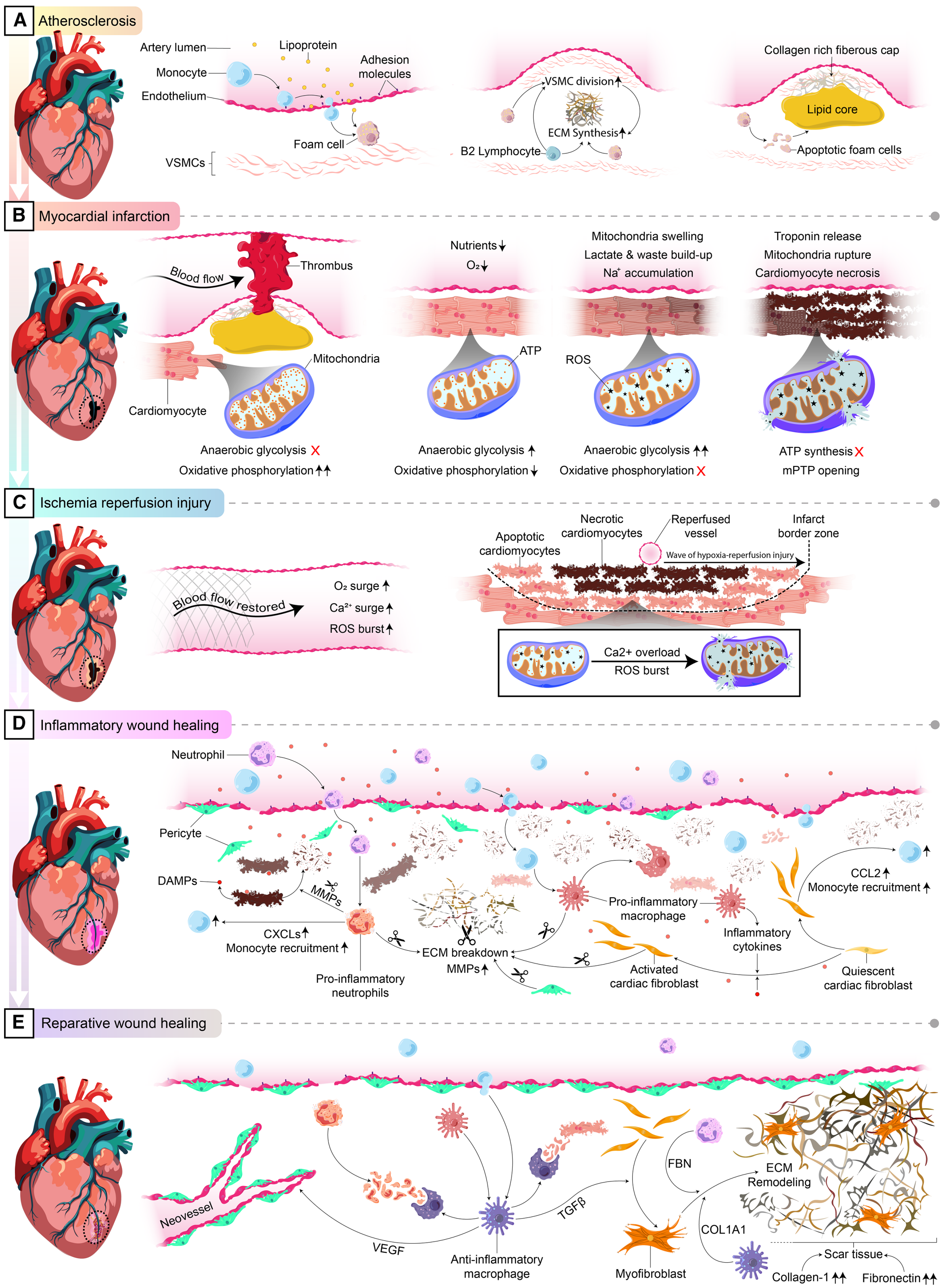

(legend on next page)

CellPress
OPEN ACCESS

CELLULAR AND MOLECULAR EVENTS BEFORE, DURING, AND AFTER ISCHEMIC INJURY

In the healthy heart, the coronary circulation sustains cardiomyocyte aerobic metabolism and contraction, while vascular and stromal cells support tissue homeostasis. Endothelial cells (ECs) lining the coronary vessels preserve barrier integrity, regulate vasomotor tone, and limit leukocyte adhesion under resting conditions.[18] Vascular smooth muscle cells (VSMCs) provide structural support, while pericytes maintain microvascular integrity and regulate vascular development.[19] Cardiac fibroblasts (CFs) help regulate extracellular matrix (ECM) homeostasis, thereby supporting normal myocardial structure and function.[20]

From coronary occlusion to acute ischemic injury

Most MIs result from disruption of an atherosclerotic plaque followed by thrombus formation in a coronary artery. Atherosclerosis is a chronic inflammatory disease of the arterial wall that develops over many years through EC dysfunction, lipid accumulation, and recruitment of circulating immune cells. Monocytes enter the vessel wall and differentiate into macrophages that take up oxidized lipids and become foam cells when uptake exceeds processing capacity.[21] Lymphocytes also contribute to the inflammatory environment promoting plaque progression. Enlarging plaques develop a lipid-rich necrotic core covered by a fibrous cap of smooth muscle cells and ECM that helps maintain plaque stability. Cap rupture or plaque erosion exposes a thrombogenic surface, triggering platelet adhesion, coagulation, and thrombus formation that abruptly reduces or stops coronary blood flow (Figure 1A).[12,22,23]

Ischemia begins when oxygen delivery to the cardiomyocytes is no longer sufficient to meet metabolic demand due to lack of blood flow.[9,12,22] This initially causes a rapid fall in oxidative phosphorylation and ATP production within cardiomyocytes, which have particularly high energy requirements. To compensate, cells increase anaerobic glycolysis, leading to lactate accumulation and intracellular acidosis. Simultaneously, reduced blood flow limits removal of metabolic by-products and disrupts ion homeostasis, contributing to impaired contractile function, mitochondrial function, and loss of membrane integrity. Dysfunctional mitochondria generate reactive oxygen species (ROS), further exacerbating cellular damage.[24,25] Although some early injury may be reversible if perfusion is restored promptly, prolonged ischemia causes irreversible cardiomyocyte death, with necrosis predominating in the infarct core (Figure 1B).[9,12,25,26] ECs and fibroblasts are also injured by ischemia, although cardiomyocyte loss dominates the acute infarct. Troponins released from damaged cardiomyocytes subsequently become detectable in the circulation and serve as key clinical biomarkers of myocardial injury.[27]

Ischemia-reperfusion injury and the early inflammatory response

Reopening the occluded coronary artery restores oxygenated blood, which is essential to halt the progression of ischemic damage, but can also trigger an additional wave of injury known as ischemia-reperfusion injury (IRI), causing further cardiomyocyte death. During early reperfusion, mitochondria that were depolarized but remained structurally intact during ischemia generate a burst of ROS as the electron transport chain is reactivated. This oxidative stress, together with restoration of physiological pH and mitochondrial Ca^{2+} overload, opens the mitochondrial permeability transition pore (mPTP). Sustained mPTP opening collapses the mitochondrial membrane potential, halts ATP synthesis, and triggers further cardiomyocyte death through both necrotic and apoptotic pathways (Figure 1C).[23–26,28]

Following IRI, the damaged myocardium enters the first stages of wound healing through a highly orchestrated inflammatory phase. Necrotic cells release damage-associated molecular patterns (DAMPs), which activate Toll-like receptors (TLRs) on neighboring cells, trigger inflammatory signaling, and promote leukocyte recruitment to the infarct. Neutrophils are among the first responders, contributing to debris clearance through matrix metalloproteinase (MMP)-mediated ECM remodeling, ROS generation, and monocyte recruitment, while also exacerbating local inflammation. Circulating monocytes are then recruited to the damaged myocardium, partly through CCL2-dependent signaling, and differentiate into pro-inflammatory macrophages that further amplify chemokine production and matrix remodeling, while clearing necrotic material.[29,30] Pericytes detach from damaged vessels, facilitating transmigration through the vessel wall, and migrate to the infarct where they proliferate. Through MMP and cytokine secretion, this promotes leukocyte infiltration, angiogenic responses, and ECM remodeling, thereby helping prepare the tissue for repair and neovascularization. CFs also adopt a pro-inflammatory phenotype in response to DAMPs and other injury-associated signals, contributing to ECM degradation and restructuring (Figure 1D).[19,23,31–33]

Inflammatory repair, fibrosis, and post-infarct wound healing

Over the following 3–7 days, the acute inflammatory response subsides, and a reparative, pro-fibrotic phase of wound healing is established. Pro-inflammatory neutrophils undergo apoptosis, while macrophages transition toward a more anti-inflammatory,

Figure 1. Schematic overview of atherosclerosis, myocardial infarction, and subsequent inflammatory and reparative responses

(A) Endothelial dysfunction promotes monocyte recruitment, foam cell formation, vascular smooth muscle cell (VSMC) proliferation, and plaque development.

(B) Plaque rupture triggers thrombus formation and coronary occlusion, leading to cardiomyocyte ischemia, metabolic failure, reactive oxygen species (ROS) accumulation, and necrotic cell death.

(C) Reperfusion restores blood flow but can induce additional injury through oxidative stress and calcium overload.

(D) The damaged myocardium initiates an inflammatory phase characterized by neutrophil and monocyte recruitment, matrix degradation, and cardiac fibroblast activation.

(E) This is followed by a reparative phase marked by macrophage polarization, neovascularization, myofibroblast activation, extracellular matrix remodeling, and formation of a fibronectin- and collagen-rich scar.

reparative phenotype, clearing apoptotic bodies through phagocytosis and releasing factors such as vascular endothelial growth factor (VEGF) and transforming growth factor β (TGF-β) that promote neovascularization and fibroblast activation. Activated fibroblasts migrate into the injured myocardium and differentiate into myofibroblasts, which deposit collagen- and fibronectin-rich ECM to form scar tissue that stabilizes the infarct and reduces the risk of rupture of the weakened myocardial wall. New vessels also sprout from the infarct border zone into the damaged tissue, supporting repair by supplying oxygen and nutrients to activated fibroblasts and myofibroblasts, facilitating matrix deposition, and limiting infarct expansion.

Over subsequent weeks and months, the reparative phase progresses to scar maturation and ventricular remodeling. Collagen fibers become increasingly cross-linked, myofibroblasts diminish or return to a more quiescent state, and many newly formed vessels regress, leaving behind a dense, collagen-rich, non-contractile scar. Although this replacement fibrosis provides structural stability, it also alters ventricular structure and mechanical properties, contributing to impaired function and compensatory hypertrophy in the surrounding viable myocardium (Figure 1E). The infarct border zone is especially important because surviving cardiomyocytes are mechanically coupled to scar tissue and experience altered load, heterogeneous repolarization, and slowed conduction, making this region prone to electrical instability, arrhythmias, and further remodeling.[31,32,34–38]

Beyond cardiomyocyte and stromal responses, autonomic nervous system dysregulation also contributes to adverse post-MI remodeling. Chronic sympathetic overactivity promotes hemodynamic stress, ventricular remodeling, arrhythmogenesis, and inflammatory activation, thereby influencing scar formation, ECM turnover, and the balance between reparative and maladaptive remodeling, while accelerating progression toward heart failure.[39]

The long-term outcome depends partly on how effectively inflammation resolves during the transition from injury to repair. Larger infarcts and persistent inflammatory signaling can prolong this process, causing chronic inflammation and excessive ECM deposition beyond the infarct. Although initial replacement fibrosis is essential, unresolved inflammation can promote reactive fibrosis, which stiffens the ventricle, disrupts electrical conduction, impairs contractile function, and increases the risk of arrhythmias and heart failure.[31,37,40] This coordinated sequence of injury, inflammation, repair, fibrosis, and electrical remodeling provides the biological framework against which preclinical MI models should be designed and evaluated.

MODELING ISCHEMIC HEART DISEASE: DOES SPECIES MATTER FOR HUMAN RELEVANCE?

Animal models have been foundational for dissecting MI mechanisms, studying post-infarct remodeling, and identifying candidate regenerative therapies. Small rodents such as mice and rats provide cost-effective, genetically tractable platforms for mechanistic studies, whereas larger animals, including pigs, sheep, and non-human primates, more closely recapitulate human cardiac anatomy, hemodynamics, and post-infarct repair, making them particularly valuable for translational and device-based studies (Figure 2A).[41–43]

However, most preclinical MI studies are performed in young, otherwise healthy animals, whereas patients typically present at older age with atherosclerosis, comorbidities, and concomitant medications. Because plaque rupture and thrombus formation are difficult to reproduce experimentally, MI is usually induced by left anterior descending coronary artery ligation, transient coronary occlusion-reperfusion, or cryoinjury.[41–45] While highly informative, each model captures only selected aspects of the pathophysiology.

Rodent and human hearts share broad anatomical and biological features, including a four-chambered structure and conserved pathways governing inflammation and wound healing. Yet they differ in several clinically relevant respects, including heart rate, ventricular wall thickness, collateral circulation, and electrophysiological and immune properties.[46–49] Rodents are therefore highly informative for dissecting pathways of cell death, inflammation, and repair but underrepresent key human MI features such as arrhythmic susceptibility, infarct expansion, border zone remodeling, inflammatory-to-fibrotic repair, and progression to chronic heart failure, potentially affecting the interpretation of therapeutic efficacy. Nonetheless, they remain an important first-line platform for testing cardioprotective interventions, typically before evaluation in larger, more costly animals. Larger animal models more accurately reproduce human coronary anatomy, ventricular geometry, infarct expansion, and border zone mechanics and are therefore preferred for studying post-MI remodeling and therapeutic interventions, despite higher cost and ethical constraints. Poor clinical translation of many interventions likely reflects limitations of experimentally induced injury models, particularly when efficacy in controlled animal studies fails to predict performance in heterogeneous patient populations (Figure 2B).[44,46,47,50]

Human induced pluripotent stem cells in cardiovascular research

Primary adult human cardiomyocytes can be studied *ex vivo*, but their limited availability, fragility, and poor long-term culture make them unsuitable for large-scale disease modeling or drug screening. Although foundational strategies for cardiac differentiation were first established using human embryonic stem cells (hESCs),[51] the advent of somatic cell reprogramming to generate human induced pluripotent stem cells (hiPSCs)[52,53] made these approaches patient-specific and more broadly applicable. hiPSCs provide a renewable human cell source that can be expanded indefinitely and differentiated into major cardiac cell types while preserving the donor genome. Directed differentiation protocols, based on modulation of developmental signaling pathways, particularly WNT signaling, now enable reproducible generation of cardiomyocytes and other cardiac cell types *in vitro*.[51,54]

These advances have enabled scalable production of hiPSC-derived cardiomyocytes (hiPSC-CMs), which exhibit human ion channel expression patterns and pharmacological responses that are generally more representative of human cardiac electrophysiology than those of rodent cardiomyocytes.[55] For example, ventricular repolarization in rodents relies on a different balance

CellPress
OPEN ACCESS

A

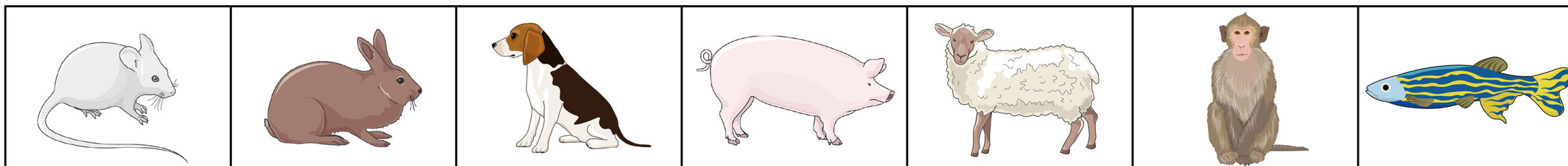

	Mouse	Rabbit	Dog	Pig/Swine	Sheep	Non-human primates	Zebrafish
**Advantages**	Low cost, fast recovery & high throughput compared to larger animal models	Low cost & closer electrophysiology to human	Closer human cardiovascular physiology over rodents	Anatomy, perfusion and healing similar to humans	Similar cardiac size and healing to humans	Among the closest model to human cardiovascular biology	Cardiac regeneration studies
**Limitations**	Small size, high heart rate, different cellular ratios & gene expression	Small size, high heart rate, fewer genetic tools	General public ethical views, legal, and cost concerns	High cost & increased risk of arrhythmias	High cost & arrhythmia sensitivity	Expense, low throughput, low availability	Structure/physiology very different, poor at modeling human MI
**Cardiac injury method**	Surgical artery ligation (permanent or transient), cryoinjury	Coronary ligation, coil embolization	Coronary ligation	Surgical ligation, balloon occlusion-reperfusion	Artery ligation, balloon occlusion-reperfusion	Surgical MI, percutaneous ischemia-reperfusion	Cryoinjury, resection

B

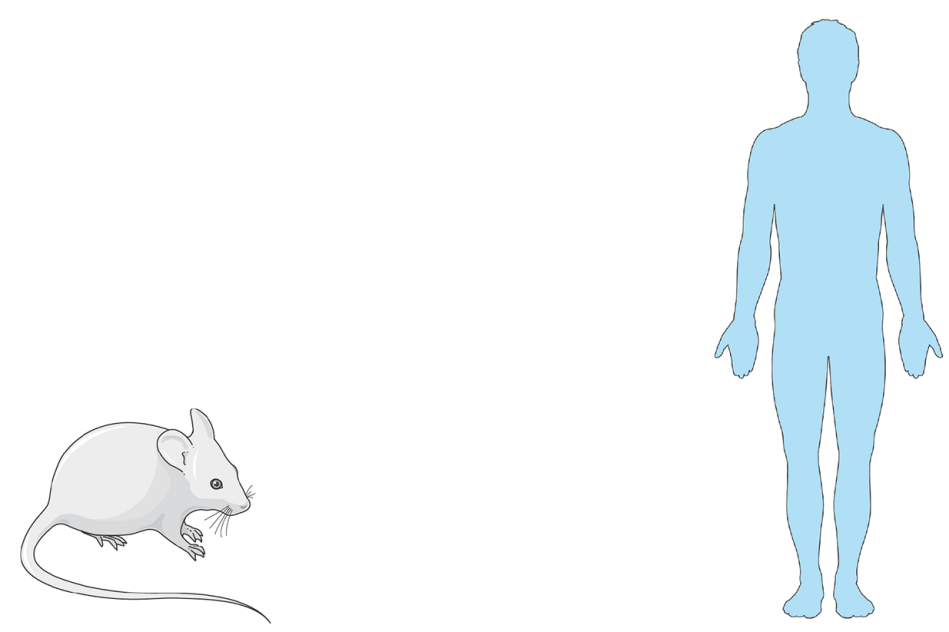

Feature	Mouse	Human	Implication
**Heart rate (beats per minute)**	400-700 bpm	60-100 bpm	Higher metabolism, affects ischemia tolerance
**Heart size & structure**	Very small heart with thin ventricular walls	Large heart with thick ventricular walls	Differences in infarct size scaling and wall stress responses
**Coronary anatomy**	Different coronary network; prominent collateral flow	Complex coronary arteries; variable collateral flow	Collateral flow influences infarct size and reproducibility
**Electrophysiology**	Short action potential duration (~50 ms), rapid repolarization	Longer action potential duration (~200-300 ms)	Mouse hearts less prone to certain arrhythmias
**Response to ischemia**	Greater tolerance to ischemic injury; faster healing timeline	More vulnerable to ischemia with larger infarcts and slow remodeling	Affects infarct evolution and healing dynamics
**Inflammatory response**	More robust and quicker inflammatory response	Slower, more complex immune response	Translational differences in inflammation-driven remodeling
**Genetic manipulation**	Well-established genetic tools for gene editing	Genetic studies limited to observational and therapeutic approaches	Allows mechanistic studies in mice not possible in humans
**Regeneration capacity**	Transient neonatal regenerative window, negligible adult regenerative capacity	Negligible regenerative capacity	Both species limited, but differences in remodeling reveal nuances

Figure 2. Animal models used to study myocardial infarction (MI) and key species differences relative to humans
(A) Overview of commonly used animal models of MI, including their major advantages, limitations, and typical methods of injury induction.
(B) Comparison of mouse and human cardiac characteristics, illustrating species differences that affect the modeling and translational relevance of MI studies.

of potassium currents than in humans, contributing to shorter action potentials.[56,57] By contrast, hiPSC-CMs express key human cardiac ion channel genes, including *SCN5A*, *CACNA1C*, *KCNH2*/hERG, and *KCNQ1*, and have therefore proved particularly useful for modeling disorders of cardiac excitability and repolarization, such as Brugada syndrome and long QT syndrome, as well as inherited cardiomyopathies.[58] Patient-derived hiPSCs also enable disease modeling in genetic backgrounds that may reflect clinically relevant aspects of the patient phenotype.[59–61] Genome editing of patient-derived or control hiPSCs can also generate isogenic pairs by introducing or correcting specific variants, thereby supporting direct investigation of causal disease mechanisms.[61–64]

Despite these advantages, standard 2D hiPSC-CM systems have important limitations. They lack the multi-cell-type composition, tissue architecture, mechanical loading, vascular interfaces, and immune components present *in vivo*,[55] although some of these features can be partially addressed by 3D and organoid-based approaches.[65,66] hiPSC-CMs also remain developmentally immature, with fetal-like morphology, sarcomere organization, calcium handling, metabolism, and electrophysiology.[67] These features limit their ability to faithfully model adult-onset cardiac diseases or predict responses that depend on mature structure, gene expression, and function.[68–71] Nonetheless, their scalability, human genetic background, and compatibility with genome engineering make hiPSC-based cardiac systems attractive for early-stage mechanistic studies, disease modeling, and higher-throughput safety and efficacy screening. These strengths and limitations are considered below in the context of MI modeling.

Positioning hiPSC-based cardiac models alongside animal models

hiPSC-based cardiac models should be viewed as complementary to, rather than replacements of, animal models. Animal models provide whole-organ and whole-body context, enabling investigation of systemic physiology, multicellular and multi-organ interactions, pharmacokinetics, and long-term remodeling after injury. In contrast, hiPSC-based systems offer genetically defined human cells suited to studying cell-intrinsic mechanisms, cell-cell interactions, and patient- or variant-specific phenotypes in a controlled setting (Table S1). Their strength lies in mechanistic dissection and more human-relevant early-stage efficacy and safety testing.

For MI modeling, hiPSC platforms should be selected and developed according to the biological question being addressed. Cardiomyocyte monolayers are useful for studying acute cell-intrinsic responses to ischemia or reperfusion, whereas multi-cell type 3D and perfused constructs are better suited to modeling tissue-level injury, repair, remodeling, and therapeutic response after MI. The following sections discuss how these platforms are being applied to model MI pathophysiology, support therapeutic screening and regenerative strategies, and advance approaches to IHD.

hiPSC MODELS OF ISCHEMIC HEART DISEASE

hiPSC-based cardiac models are becoming increasingly valuable for investigating MI by providing human cellular systems that help address species-dependent differences in cardiac electrophysiology and metabolism while preserving human genetic backgrounds. Figure 3 highlights representative hiPSC-based *in vitro* platforms used to model aspects of MI and IRI. These range from simple 2D cultures to more complex organoids, engineered heart tissues (EHTs), and microphysiological heart-on-chip systems. Together, these models capture different features of MI pathophysiology, from cardiomyocyte death to fibrotic remodeling and electrical dysfunction, although none reproduce the full spatial and temporal complexity of the disease (Figure 3; Table S2).

Across these platforms, MI-related phenotypes are induced according to the disease phase and model format. Acute ischemia and IRI are typically simulated using hypoxia, nutrient or glucose deprivation, chemical ischemia, or flow interruption followed by reoxygenation, whereas focal tissue injury can be introduced by cryoinjury. Later remodeling and fibrosis are more commonly modeled using TGF-β1 stimulation, altered cell-type ratios, matrix stiffening, or prolonged culture under stress conditions.

2D hiPSC-CM models

Early studies primarily employed 2D hiPSC-CM cultures, which provide accessible and scalable platforms for dissecting cardiomyocyte-intrinsic responses to ischemia and reperfusion. Injury is typically induced by chemical ischemia or by exposing cells to hypoxia (0%–1% O_2) together with metabolic stress, such as glucose deprivation, followed by normoxic reoxygenation.[72–74] These approaches reproduce key features of acute IRI and cardiomyocyte death. For example, Häkli et al. used hypoxic and nutrient-altered conditions to induce ischemic cardiomyocyte death through mitochondrial dysfunction, thereby mimicking aspects of MI *in vitro*.[72] Similarly, Hidalgo et al. reported that metabolically matured hiPSC-CMs, cultured in galactose- and fatty-acid-containing medium to enhance oxidative metabolism, were more sensitive to severe acidosis, mirroring the metabolic vulnerability of the adult myocardium during IRI.[73]

Although 2D models enable controlled analysis of acute cardiomyocyte injury after ischemic or reperfusion-like stress, they remain constrained by hiPSC-CM developmental immaturity. As a result, subtle changes in contractility, electrophysiology, or metabolism may fall below the threshold of assay detection.[75] The absence of non-cardiomyocyte cell types also means indirect effects mediated by fibroblasts, ECs, or immune cells are not captured. Their lack of 3D tissue architecture, physiological mechanical loading, and native stromal cell diversity means these 2D systems primarily model the early, cell-autonomous phase of IRI rather than the subsequent multicellular processes driving inflammation, fibrosis, and tissue remodeling.

3D microphysiological models

To address some limitations of 2D cardiomyocyte-only cultures, 3D and microphysiological hiPSC-based systems have been developed to model myocardial injury and downstream consequences. These include engineered heart tissues (EHTs),[76] cardiac spheroids,[77] multi-cell type cardiac microtissues,[78] self-organizing cardiac organoids/cardioids,[65,66] and heart-on-chip (HoC) platforms,[79] many of which now benefit from improved

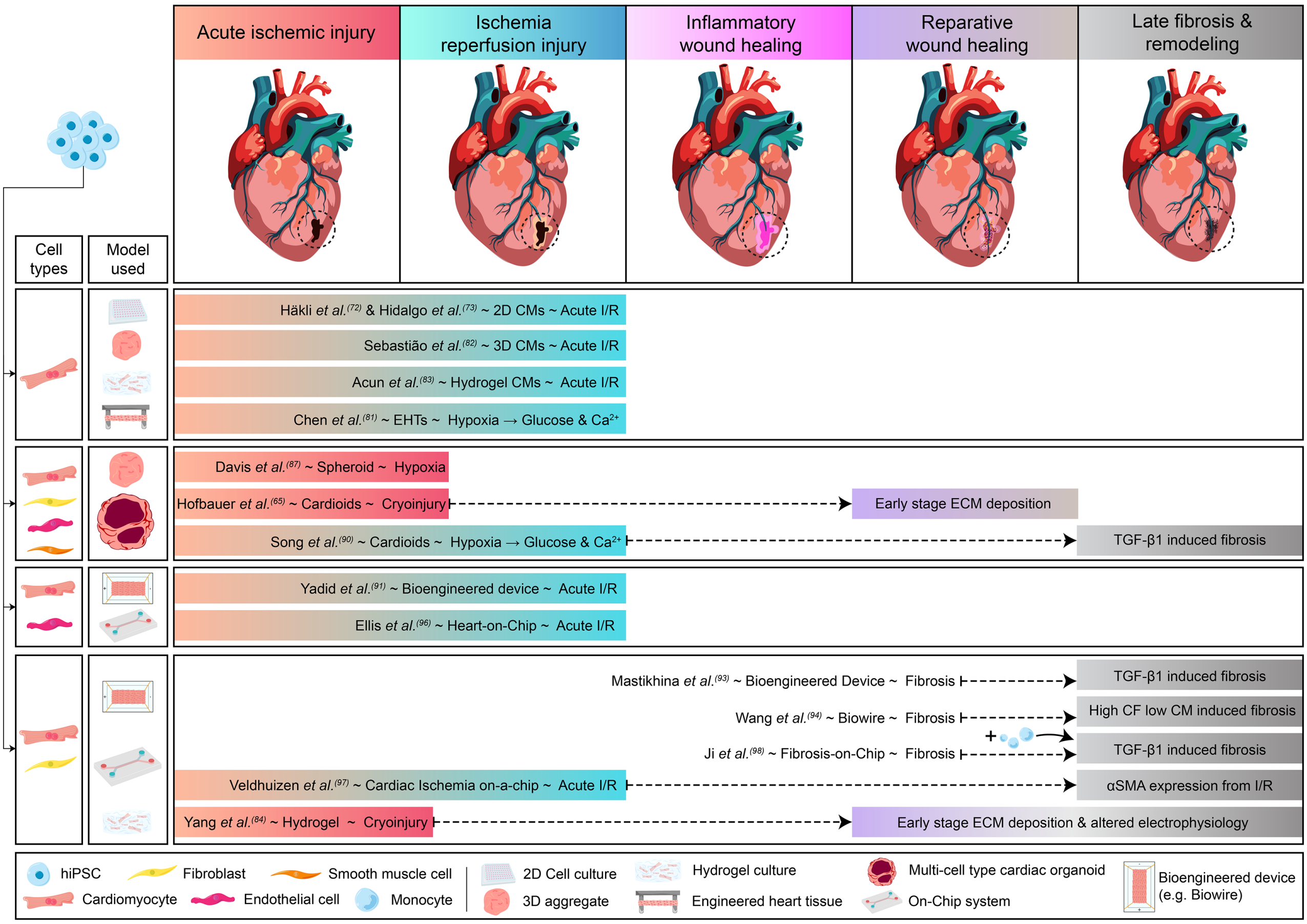

Figure 3. hiPSC models of MI and the stages of disease they capture

Stages of MI are depicted, including acute ischemic injury (red), ischemia-reperfusion injury (blue), inflammatory wound healing (pink), reparative wound healing (purple), and late-stage fibrosis/remodeling (gray). The figure summarizes representative hiPSC studies, indicating the cell type(s), model format, and aspects of MI pathophysiology captured by each model.

standardization and scalability.[80] Compared with 2D systems, these models better capture local differences in oxygen, nutrient, and metabolite exposure across the tissue, as well as multicellular interactions and mechanical or electrophysiological function, thereby enabling investigation of later and more integrated aspects of MI pathophysiology.

In EHTs, hiPSC-CMs, often combined with fibroblasts, are embedded within fibrin- or collagen-based matrices that organize into elongated tissues and generate contractile force by pulling against flexible pillars or posts.[76] EHTs can reveal injury-associated changes in force generation, ECM remodeling, and tissue stiffness that are not readily captured in 2D cultures. For example, Chen and Vunjak-Novakovic showed contractile dysfunction in hiPSC-CM-only EHTs following IRI, with contractions halting after reperfusion.[81] Single-cell-type bioreactor-based cardiac aggregates have also been used to impose controlled cycles of hypoxia and reperfusion, revealing load-dependent contractile failure and progressive matrix stiffening.[82] Hydrogel systems can also mimic physiological tissue mechanics. Acun et al. showed that prolonged tissue maturation increased cardiomyocyte susceptibility to IRI, linking *in vitro* aging with the greater vulnerability of aged human myocardium.[83] Yang et al. combined hiPSC-CMs and hiPSC-CFs in a hydrogel, induced acute damage using cryoinjury, and monitored early ECM deposition and fibroblast-mediated wound healing.[84] This led to altered cardiomyocyte electrophysiology and captured several features of MI progression.

Compact 3D cardiac spheroids, formed by combining pre-differentiated cells, can similarly exploit size-dependent oxygen gradients that develop within aggregates larger than 500 μm, generating endogenous hypoxic zones under low oxygen conditions.[85] Using this principle, Sharma et al. established a stepwise oxygen modulation protocol in cardiac spheroids that elicited cardiomyocyte-specific death, contractile decline, and transcriptomic changes consistent with ischemic injury.[86] Richards et al. likewise used hiPSC-derived cardiac spheroids to show that spontaneous hypoxic cores are exacerbated by norepinephrine exposure, resulting in infarct-like hypertrophy and ROS accumulation.[87] These systems provide versatile platforms for mechanistic studies and, in some cases, drug screening under more physiologically relevant stress conditions.

Further complexity is achieved in multi-cell-type cardiac microtissues and self-organizing organoid systems. Cardiac microtissues assembled from pre-differentiated hiPSC-CMs, hiPSC-CFs, and hiPSC-ECs show improved cardiomyocyte maturation and scalability for functional compound screening.[78,88] A related 3D cardiac microtissue model generated by aggregating 2D-differentiated cardiovascular cells was reported to maintain multiple cardiac cell types and undergo structural and metabolic maturation during long-term 3D culture.[89] By contrast, self-organizing cardioids and epicardioids capture aspects of cardiac developmental organization and tissue architecture, including cavity-like structures and epicardial layer formation.[65,66] Hofbauer et al. applied focal cryoinjury to human cardioids, generating localized necrotic lesions that triggered ECM deposition and partial functional recovery reminiscent of early scar formation *in vivo*.[65] Song et al. generated multi-cell-type hiPSC-derived heart organoids approximating the composition of the adult ventricle and used a two-step chemical ischemia-reperfusion protocol combining cobalt chloride and glucose deprivation, followed by reoxygenation with high-glucose and calcium-rich medium.[90] This resulted in HIF-1α activation, cell death, sarcomere disruption, and inflammatory responses consistent with acute MI. Following TGF-β1 exposure after reperfusion, these organoids developed fibrosis, conduction slowing, altered electrophysiology, and tachyarrhythmia, linking acute injury to later fibrotic and electrical remodeling within a single platform.[90]

Microfabrication and tissue-engineering approaches have also been combined with hiPSC models to create bioengineered cardiac devices with improved cardiomyocyte maturation and functional readouts compared with 2D cultures. Yadid et al. showed that hiPSC-CMs on film cantilevers subjected to IRI were rescued by extracellular vesicles secreted from ECs.[91] Other approaches include the Biowire system, in which aligned cardiomyocytes are generated between conductive wires and show enhanced maturation after electrical pacing.[92] TGF-β1 exposure[93] or high cardiac fibroblast-to-cardiomyocyte ratios[94] have been used in Biowire-based models to study late-stage cardiac fibrosis.

A promising approach for advancing bioengineered cardiac models is organ-on-chip and HoC technology, which incorporates microfluidics and perfusable vascular networks to provide more physiologically controlled systems for modeling CVD.[79,95] For example, a HoC platform developed by Ellis et al. captured aspects of endothelial barrier dysfunction and microvascular injury alongside cardiomyocyte death by temporarily halting oxygen supply and flow through the microfluidic system, recapitulating key features of IRI.[96] HoC models of later-stage fibrosis have also been established through IRI[97] or TGF-β1 gradient exposure with the introduction of hiPSC-derived macrophages,[98] enabling study of conduction slowing, proarrhythmic behavior, and reduced contractility associated with chronic fibrotic remodeling.[79,94] Together, these 3D and microphysiological systems extend hiPSC-based MI modeling beyond acute cell death toward tissue-level injury, repair, and dysfunction.

Readouts and alignment with human MI

Because hiPSC-based MI models capture defined components of the disease rather than the complete patient-level syndrome, their value depends on selecting readouts that match the biological phase being modeled. Acute ischemia-reperfusion models require endpoints reporting cardiomyocyte injury, metabolic failure, calcium dysregulation, electrophysiological instability, and early contractile decline. In contrast, models designed to study later repair, fibrosis, or remodeling require readouts of fibroblast activation, ECM deposition, tissue stiffening, conduction slowing, and longer-term functional impairment. Alignment with human MI therefore depends less on reproducing the entire clinical diagnosis *in vitro* and more on linking models to a defined context of use (CoU) and clinically relevant disease features.

Clinically, the Fourth Universal Definition of MI distinguishes myocardial injury from myocardial infarction. Acute myocardial injury is defined by cardiac troponin values above the 99th percentile upper reference limit, together with a rise and/or fall on serial testing. Acute MI requires additional evidence of myocardial ischemia, such as symptoms consistent with ischemia, new ischemic ECG changes, imaging evidence of new loss of viable myocardium or regional wall-motion abnormality, or identification of a coronary thrombus.[99] These clinical criteria cannot be transferred directly to isolated cells or engineered tissues but provide useful anchors for aligning *in vitro* readouts with major diagnostic and pathophysiological features of MI.

At the level of cell and tissue injury, hiPSC models can measure cardiomyocyte damage using biomarkers and viability assays. Secreted cardiac troponins, CK-MB, LDH, myoglobin, and exosomal miRNAs provide injury-associated readouts that can be sampled periodically in culture medium.[26,80,90,100] These measurements are most informative when interpreted dynamically, for example, by assessing whether injury and reoxygenation induce a time-based increase in biomarker release relative to matched uninjured controls, rather than attempting to reproduce clinical diagnostic thresholds. Complementary acute cell death assays include LDH release, propidium iodide (PI), or combinations of ethidium homodimer and Hoechst staining to distinguish live and dead cell populations. The mode of cell death can be further characterized using TUNEL, Annexin V, or cleaved caspase-3 staining. Combined with general cell death dyes, such as PI or 7-AAD, these approaches can help distinguish apoptotic from non-apoptotic cell death.[101]

Mitochondrial and metabolic readouts are also central to modeling MI and IRI. In the adult heart, ischemia and reperfusion disrupt oxidative phosphorylation, alter substrate use, increase ROS generation, and impair mitochondrial membrane integrity.[25,102] These changes are accompanied by acute energetic stress, including reduced ATP production, increased reliance on glycolysis, lactate accumulation, and impaired recovery of oxidative metabolism after reperfusion. In hiPSC-based systems, these processes can be assessed using Seahorse-based metabolic flux analysis measuring glycolytic activity and oxygen consumption, assays for ATP depletion or recovery, and fluorescent reporters of mitochondrial membrane potential or ROS, such as MitoTracker CMXRos and MitoSOX.[103] These assays provide a mechanistic link between *in vitro* injury and key metabolic features of human IRI, although interpretation must account for the immature metabolic state of hiPSC-CMs.

CellPress
OPEN ACCESS

Functional readouts also provide opportunities to align hiPSC models with clinical features of MI. In patients, cardiac function is assessed non-invasively by echocardiography and cardiac MRI, which quantify left ventricular ejection fraction, regional wall motion, myocardial strain, infarct size, and microvascular obstruction.[104] *In vitro*, hiPSC-CM contractile and electrophysiological changes can be assessed using multi-electrode arrays (MEAs), patch clamp, voltage or calcium imaging, and contraction analysis. These approaches can detect altered field potential duration, slowed conduction, abnormal repolarization, reduced calcium transient amplitude, delayed calcium decay, and arrhythmic events.[71,88] This is particularly relevant because acute ischemia and post-infarct remodeling are strongly associated with electrical instability and ventricular arrhythmias.[38] A hiPSC-CM-on-chip model subjected to hypoxia reproduced pro-arrhythmic features, including irregular calcium transients, abnormal beating patterns, and altered contractile amplitudes,[105] illustrating how hiPSC systems can investigate ischemia-induced arrhythmias and candidate therapeutics. Contractile function can also be measured through pillar or cantilever deflection in EHTs and Biowire systems or video-based pixel displacement and pseudo-force calculations using tools such as MUSCLEMOTION and CardioMotion.[76,106]

Molecular profiling can further align hiPSC models with specific phases of MI pathology. Gene expression profiling, proteomics, and spatial or single-cell approaches can assess stress, inflammatory, and fibrotic responses that reflect aspects observed in human MI.[100] Relevant markers include genes and proteins associated with mitochondrial stress (e.g., *BAX* and *NOX4*), inflammatory signaling (e.g., TGF-β, nuclear factor κB (NF-κB), and JNK pathway components), and fibrotic responses (e.g., *COL1A1*, *FBN1*, *ACTA2*, and MMPs). These endpoints are useful in multicellular 3D models that aim to capture matrix remodeling, fibroblast activation, conduction slowing, or scar-like tissue responses. However, because many models investigate only one component of the post-MI response, such as acute cardiomyocyte injury or TGF-β-induced fibrosis, molecular readouts should be interpreted in relation to the specific phase of MI being modeled.

Overall, alignment between hiPSC models and human MI should be based on predefined contexts of use. A model intended to study acute IRI should focus on injury biomarkers, mitochondrial dysfunction, electrophysiology, calcium handling, and early contractile decline. A model intended to study post-infarct fibrosis should instead focus on fibroblast activation, ECM deposition, conduction slowing, and longer-term contractile impairment. Combining biomarker, metabolic, electrophysiological, contractile, and molecular endpoints provide a stronger basis for comparing hiPSC systems with clinically relevant features of MI, while acknowledging that they capture selected windows of the disease rather than the complete pathophysiology.

Current limitations and priorities for improving hiPSC models of MI

Although context-specific readouts can strengthen alignment between hiPSC models and human MI, model fidelity remains constrained by biological and technical limitations. A central issue is the maturation state of hiPSC-CMs. Adult cardiomyocytes rely predominantly on oxidative metabolism and fatty acid oxidation, whereas hiPSC-CMs retain a more fetal-like phenotype, with greater reliance on glycolysis, limited oxidative capacity, residual proliferative potential, and contractile properties that differ from mature human cardiomyocytes. This immaturity can alter the calcium dysregulation, contractile dysfunction, and downstream remodeling responses that characterize ischemic injury *in vivo*. It is particularly relevant for MI modeling because ischemia, reperfusion, and the surviving border zone are associated with major changes in substrate use and mitochondrial function.[102] Since hiPSC-CMs have a relatively immature metabolic state, they may not fully capture disease-associated metabolic shifts or their downstream consequences. Metabolic maturation strategies, including substrate switching, prolonged culture, and biomechanical or electrical conditioning, can improve oxidative capacity and contractile performance but still do not fully reproduce the adult human cardiomyocyte phenotype.[68–71,73,107]

Another limitation is cardiomyocyte subtype and regional identity. Many hiPSC models, particularly 2D systems, rely on cardiomyocyte populations generated through optimized differentiation protocols that are often described as ventricular-like but remain heterogeneous and incompletely specified. While useful for targeted mechanistic studies, this does not fully capture the regional diversity of the native myocardium, including differences between ventricular subtypes, conduction system cells, pacemaker cells, and cardiomyocytes from distinct developmental heart fields. These distinctions are relevant to MI because infarction, border zone remodeling, conduction slowing, and arrhythmia susceptibility are influenced by regional tissue identity and electrophysiological heterogeneity. More recent differentiation strategies can generate specific cardiomyocyte subpopulations, including sinoatrial pacemaker-like cells,[108] although these are yet to be incorporated into MI models. Assembloid-based cardiac organoid systems combining cardiomyocytes from different heart field origins are beginning to address this limitation and may improve the developmental and physiological relevance of future models.[109,110]

Beyond cardiomyocyte maturity and subtype identity, most hiPSC-based MI platforms lack the full complement of non-cardiomyocyte cell types that coordinate wound repair *in vivo*. Among the most critical omissions are immune cells, which regulate the inflammatory and reparative phases of MI, including debris clearance, inflammation resolution, angiogenesis, matrix remodeling, and scar maturation.[29,30] Although some platforms have begun to incorporate hiPSC-derived macrophages or generate macrophage-like populations during organoid differentiation,[111,112] immune cell integration remains underexplored in hiPSC-based MI models. Future models should capture not only immune cell presence but also recruitment, activation state, interactions with cardiac cells, and contributions to inflammation resolution and repair. Incorporating human blood-derived immune cells may offer a complementary strategy for addressing this gap.

Other supporting cardiac cell types are also frequently absent or incompletely represented in hiPSC MI models. Fibroblasts, epicardial cells, ECs, VSMCs, and pericytes all contribute to post-MI inflammation, ECM remodeling, neovascularization,

vessel stability, and tissue repair.[18–20,34] Although several hiPSC cardiac models now incorporate fibroblasts and ECs,[65,74,86,87,90] the relative abundance, maturation state, spatial organization, and injury-induced phenotypic transitions of these populations often remain poorly controlled. As a result, many models capture selected paracrine or structural interactions but do not reproduce the coordinated multi-cell-type repair program occurring *in vivo*. Autonomic innervation is another underrepresented component. Human autonomic neurons and innervated cardiac microphysiological systems have been generated[108,113] but remain largely exploratory and have not been adapted to model MI injury, infarct healing, or post-infarct remodeling directly.

Although vascularization of engineered cardiac tissues is advancing, it remains a major bottleneck. ECs can self-organize into vessel-like structures in cardiac tissues and organoids,[65,78] but these networks are often sparse, immature, non-perfused, and lack the hierarchical branching and mural cell support of the human coronary microvasculature. ECs alone are therefore insufficient to replicate the complex vascular organization of the human myocardium. Emerging vascularized organ-on-chip systems are beginning to address this limitation, at least for disease modeling applications.[95] Perfusion can also promote vessel development and tissue viability,[114] although it is not yet feasible or standardized across all model formats. Including immune cells may further support vascular maturation and stabilisation,[115] suggesting that several cell populations missing from many MI models are functionally interdependent.

The size and architecture of 3D cardiac constructs create additional limitations. Larger spheroids, organoids, and EHTs can develop oxygen and nutrient gradients, which may lead to hypoxic or necrotic regions even without an intended injury stimulus.[85] This is particularly important for MI modeling because uncontrolled baseline hypoxia can confound interpretation of experimental ischemia or reperfusion injury.[116] Additionally, most 3D hiPSC models do not reproduce the ordered myocardial architecture of the adult ventricle. In the heart, cardiomyocyte orientation changes across the ventricular wall, and the aligned, laminar organization of the myocardium contributes to wall thickening, torsion, force transmission, and direction-dependent electrical conduction. During MI, this architecture is disrupted by cardiomyocyte death, edema, microvascular injury, matrix deposition, and scar formation, contributing to impaired contraction and remodeling.[117] Although engineered alignment strategies can reproduce some aspects of myocardial anisotropy, current hiPSC models still lack the complex organization of the human heart.

Increasing cellular and structural complexity also introduces practical challenges. Complex platforms are often more difficult to produce reproducibly, require more extensive quality control, and can complicate interpretation because multiple cell types and injury responses change simultaneously. Microphysiological and HoC platforms provide greater control over perfusion, oxygen gradients, biomechanical cues, and spatial patterning but introduce constraints related to device fabrication, throughput, long-term culture stability, assay standardization, and compatibility with downstream molecular or high-content analyses. This reinforces the need to match model complexity to the biological question, with readouts selected according to the intended CoU. Rather than aiming for a single model that recapitulates all aspects of MI, the field will likely require a portfolio of complementary hiPSC systems. Used in this way, hiPSC models can complement animal models and human clinical data, providing increasingly precise human systems for studying ischemic injury, remodeling, and therapeutic response.

TRANSLATION AND THERAPEUTIC APPLICATIONS

Beyond modeling MI pathophysiology, hiPSC-based cardiac systems are increasingly used for therapeutic discovery, predictive testing, patient stratification, and regenerative repair. These applications extend hiPSC technologies into translational workflows in which human-relevant cardiac platforms can identify candidate therapies, assess safety liabilities, and evaluate strategies for repairing the injured heart.

hiPSC cardiac models for drug screening and therapeutic discovery

As fidelity and scalability of hiPSC-based cardiac models improve, so too do their translational applications in drug discovery. One of the clearest areas of progress is cardiac safety pharmacology, where human-relevant *in vitro* systems are increasingly used alongside, and in some contexts instead of, traditional animal-based approaches.[118] Regulatory support has also increased, with the Food and Drug Administration (FDA) Modernization Act 2.0 and subsequent FDA initiatives supporting scientifically valid alternative methods, including cell-based, organ-on-chip, and *in silico* systems.[119,120] The European Medicines Agency (EMA) also supports the regulatory use of new approach methodologies (NAMs) where scientifically justified. In particular, the CiPA (Comprehensive *in vitro* Proarrhythmia Assay) and JiCSA (Japan iPS Cardiac Safety Assessment) initiatives have shown that hiPSC-CM platforms can contribute to classifying compounds by proarrhythmic risk, supporting their value in non-animal cardiac safety testing.[121–124]

However, conventional 2D hiPSC-CM cultures are limited in their ability to capture tissue-level contractile responses, multicellular interactions, and force-dependent pharmacology. 3D EHTs improve physiological relevance, but early implementations required high cell numbers and specialized instrumentation, increasing costs and restricting throughput.[76] More recently, miniaturized EHT formats have reduced cell requirements by up to 40-fold and can be implemented in multiwell plates compatible with higher-throughput workflows.[125] Other scalable 3D formats include scaffold-free cardiac microtissues assembled from pre-differentiated hiPSC-CMs, hiPSC-CFs, and hiPSC-ECs in 384-well formats.[88] These microtissues show stable electrophysiological and contractile properties and can identify positive and negative contractile compound responses that are not well captured in 2D cultures.[78,88] Automation has further improved the consistency and throughput of tissue generation and drug-response profiling, moving complex 3D cardiac systems toward practical screening applications (Figure 4A).[88,121,126,127]

At present, high-throughput hiPSC cardiac screening focuses mainly on cardiac safety, electrophysiology, and functional pharmacology. Application of these platforms to ischemic injury and cardioprotective drug discovery remains less advanced, with

CellPress
OPEN ACCESS

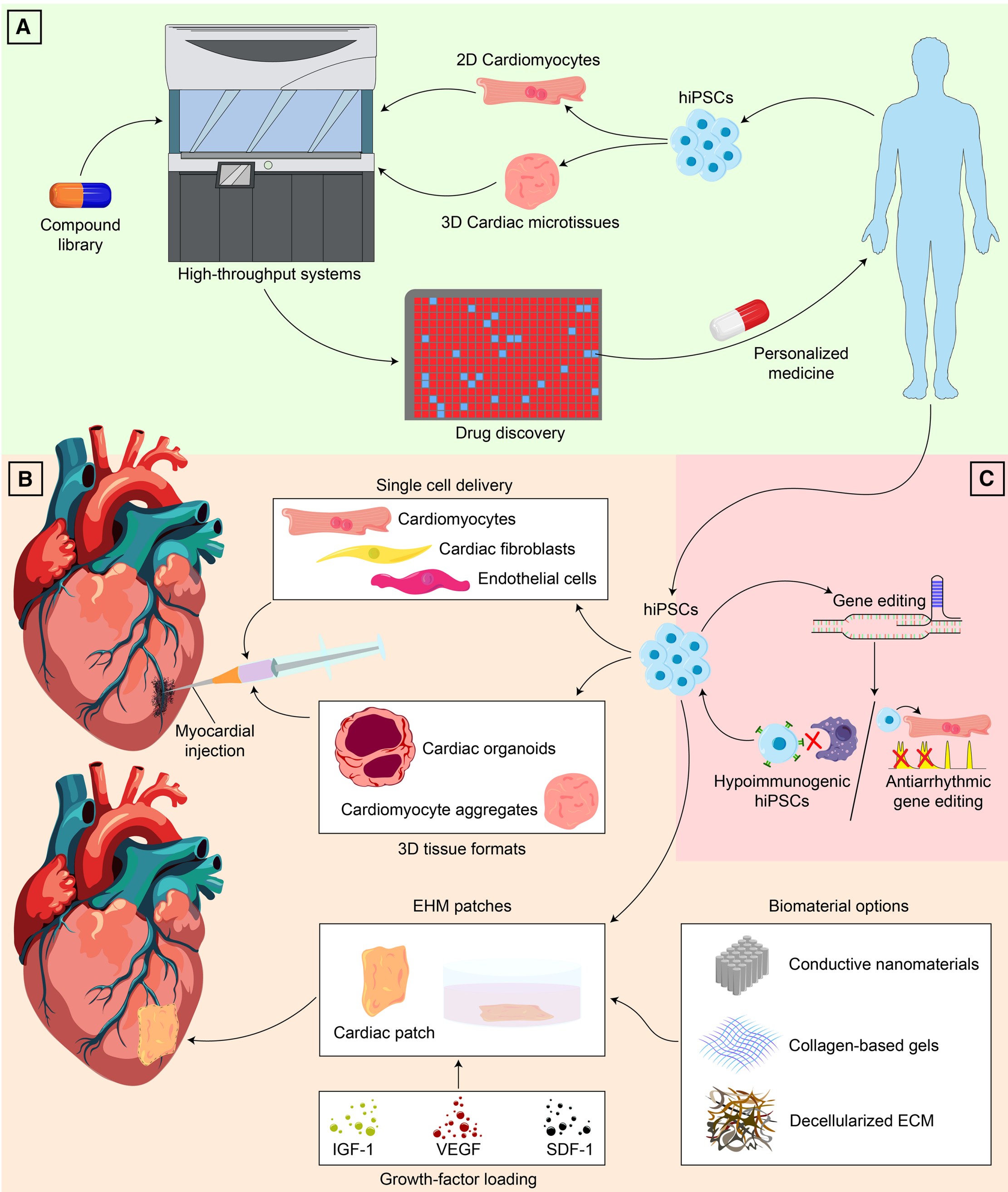

Figure 4. Translational applications of hiPSC-based cardiac models in therapeutic discovery and cardiac repair

(A) Patient-derived hiPSCs can be differentiated into cardiomyocytes, which can be used in 2D or assembled with other differentiated cardiovascular cell types into cardiac microtissues for high-throughput drug screening. These systems can support early-stage drug discovery, functional pharmacology, and personalized medicine approaches (green, currently feasible).

(B) hiPSCs can also be differentiated into cardiac cells and tissues for regenerative applications. These may be delivered as single cells, multicellular aggregates, cardiac organoids, or engineered tissues directly to the injured myocardium or assembled into cardiac patches for epicardial implantation. Patches may incorporate different biomaterials and growth factors to improve integration and stability (orange, under clinical evaluation).

(C) Genome engineering can generate hypoimmunogenic hiPSC lines as potential universal donor or other allogeneic cell sources for future regenerative therapies. Editing ion-channel and automaticity-related pathways may also reduce graft-associated arrhythmia risk after cardiomyocyte transplantation. Such approaches could reduce immune rejection and the need for immunosuppression, while improving the safety of hiPSC-based cardiac therapies (red, future possibilities).

current studies providing proof of concept rather than established screening workflows.[84,87] For example, Gaballah et al. used a hiPSC-based ischemia-on-chip model to evaluate levosimendan and its active metabolite OR-1896.[128] Treatment preserved cellular morphology, improved calcium handling, and reduced injury marker release, supporting the utility of such models for cardioprotective drug testing. De Korte et al. established an automated 384-well workflow for generating 3D cardiac microtissues and performed high-throughput antiarrhythmic screening of 2,112 compounds.[88] In parallel, Zhang et al. used hiPSC-CFs for large-scale compound screening, with hits counter-screened in hiPSC-CMs and hiPSC-ECs to exclude cardiotoxicity before validation in EHTs.[129] Together, these examples illustrate how hiPSC cardiac systems can support therapeutic drug discovery by linking scalable primary screening with multi-cell-type validation and candidate prioritization for further testing. Progress in this area will require continued refinement of injury models, assay robustness, and fit-for-purpose multicellular model design.

Patient-specific modeling and *in silico* integration

An important translational strength of hiPSC-based cardiac models is their ability to capture patient- and donor-specific biology in controlled experimental settings. This was first established in inherited arrhythmia and cardiomyopathy models, where patient-derived hiPSC lines reproduced hallmark phenotypes such as prolonged repolarization, altered calcium handling, and contractile dysfunction. Genome-edited isogenic hiPSCs have further linked these phenotypes directly to specific variants when the genetic background is controlled.[62–64]

Cardio-oncology provides clear examples of this patient-specific predictive value. hiPSC-CMs from patients who developed doxorubicin-induced cardiomyopathy showed greater cell death, mitochondrial dysfunction, calcium dysregulation, and oxidative stress than those from more tolerant patients, with similar observations reported for trastuzumab exposure.[130,131] Although not MI-specific, these studies support the broader concept of "clinical trials in a dish," in which patient-derived cells are used to stratify risk and identify differential treatment responses.[127,132]

In silico approaches, including digital twin platforms, provide a complementary route for integrating hiPSC-based experimental data with clinical and mechanistic models. These approaches can combine patient-specific clinical data with experimentally derived measurements of electrophysiology, contraction, metabolism, or injury response to support predictions of cardiac function and treatment response.[133] Application of these concepts to IHD remains underdeveloped. Patient-specific hiPSC models of MI or IRI could help identify individuals at high risk of adverse outcomes or predict differential responses to cardioprotective therapies.

hiPSC-based systems may also help address sex-related differences in MI and IHD. Clinical presentation, injury patterns, and outcomes differ between men and women, and hiPSC models allow contributions from sex chromosomes, sex-biased gene expression, and hormonal milieu to be examined under controlled conditions. Recent work suggests hiPSC-CMs retain intrinsic sex dimorphism even without exogenous hormones, with female-derived cells differing in calcium handling, beat regularity, hypoxia-related gene expression, and electrophysiological properties compared with male hiPSC-CMs.[134,135] Although this area remains underexplored, hiPSC models could help connect population-level sex differences in MI and IHD with mechanistic studies of ischemic injury, remodeling, and therapeutic response.

Progress in regenerative applications: hPSC-derived cardiac repair

Regenerative repair is another important translational application of hiPSC-based cardiac platforms. Although detailed coverage of cardiac cell therapy is beyond the scope of this review, recent reviews have described how hiPSC-CMs and engineered tissues are being developed to restore function after MI.[136–140] These strategies broadly involve either direct delivery of cells or cell aggregates to the injured myocardium or implantation of preformed engineered tissues.

Direct delivery of hiPSC-CMs is a straightforward approach to remuscularize the infarcted ventricle but remains limited by poor retention, variable engraftment, and the need to deliver large numbers of viable cells to damaged tissue. These issues can be partially mitigated by combining cells with supportive biomaterials, such as fibrin,[141] or transplanting multicellular aggregates rather than dispersed cardiomyocytes.[142–144] In preclinical animal models, such strategies have improved graft survival, vascularization, and functional benefit, particularly when stromal cell types such as fibroblasts, ECs, and pericytes are included alongside cardiomyocytes.[141,145]

An alternative and increasingly prominent strategy is the use of engineered heart muscle (EHM), similar to the EHTs described above, generated from hiPSC-derived cardiac cells and assembled into patch-like constructs. Placed over the epicardial surface, these constructs are intended to provide mechanically integrated, force-generating tissue over the injured region. Compared with direct cell injection, patch-based constructs may offer improved cell retention and structural support and may reduce arrhythmia risk associated with dispersed grafted cardiomyocytes, although this remains under investigation. In Japan, a first-in-human case of hiPSC-CM patch transplantation for severe ischemic cardiomyopathy was reported in 2022.[146] More recently, EHM allografts were shown to remuscularize and improve function of chronically injured hearts in rhesus macaques.[147] That study also provided early human proof of concept with grafted cardiomyocyte retention, organized sarcomeres, and host capillary infiltration confirmed following explantation of the transplanted heart.[136,147]

As of 2025, seven hPSC-derived cardiac therapies were undergoing clinical evaluation.[148] Several programs are evaluating direct delivery of hPSC-CMs, including intramyocardial injection approaches such as HiCM-188,[149] whereas others focus on engineered-tissue or patch-based products.[148] The BioVAT-HF program has reported early results from an open-label phase I/II study in which engineered heart muscle allografts were transplanted onto the epicardial surface of patients with advanced heart failure, with evidence of increased target-wall thickness and preliminary improvements in cardiac function.[150] In addition, the allogeneic hiPSC-derived cardiac patch therapy RiHeart received conditional approval in Japan in 2026.[146] Although

long-term efficacy, durability, and safety remain to be established, these developments show that hPSC-derived cardiomyocyte therapies are progressing clinically through both direct cell-delivery and engineered-tissue approaches.[136,148]

In addition to their therapeutic use, hPSC-based systems can support regenerative medicine by identifying candidate repair pathways. For example, a recent study using human cardiac organoids in a mouse MI model identified clusterin (CLU) as a cardioprotective and regenerative factor that promoted reparative macrophage polarization and cardiomyocyte proliferation.[151] *CLU* overexpression, either by gene delivery or transplantation of human CLU-enriched organoids, reduced scar formation and improved contractile function, illustrating how hPSC-based cardiac models can uncover candidate pathways for myocardial repair.

Despite progress, several major challenges remain for hPSC therapeutics, including long-term graft survival, immune compatibility, electrical integration, arrhythmia risk, large-scale manufacturing, and durable functional benefit. In particular, the electrophysiological consequences of transplanting cardiomyocytes remain an active area of investigation. Graft-derived automaticity can contribute to ventricular arrhythmias after cardiomyocyte cell therapy, and genome editing of excitatory- and pacemaker-related ion channels can reduce this risk,[152] although not all anti-automaticity engineering strategies are sufficient.[153] EHM patch approaches may offer a different electrophysiological profile but require continued evaluation in clinically relevant settings. Regenerative hiPSC- and hESC-based therapies for MI and ischemic cardiomyopathy are therefore advancing from proof of concept toward early clinical translation but remain emerging rather than established therapeutic modalities.

Integration of biomaterials, gene editing, and bioengineering

Building on these regenerative approaches, efforts are increasingly focusing on engineering the graft itself to improve retention, survival, integration, and function after transplantation. This includes tailored biomaterials, scaffold architectures, growth-factor delivery, and cell engineering strategies to enhance vascularization, reduce immune rejection, and optimize electrophysiological properties. Together, these approaches shift hiPSC-based cardiac repair from simple cell delivery toward more precisely engineered therapeutic systems.

A range of scaffold materials and architectures has been explored to improve the performance of implanted cardiac tissues. Conductive nanomaterials have been incorporated into engineered tissues to improve electrical coupling and conduction properties both within the graft and potentially at the graft-host interface.[154] In parallel, materials including collagen-based hydrogels and decellularized ECM can be tuned in stiffness, anisotropy, and degradation rate to better match myocardial biomechanics and promote aligned tissue organization.[155–157] A complementary biomaterial approach was described by Sridharan et al., who used aligned coaxial polycaprolactone-gelatin electrospun patches seeded with hiPSC-CMs in a porcine MI model.[158] These bioengineered patches supported durable cell engraftment, improved left ventricular ejection fraction, and reduced scar size, illustrating the potential of engineered scaffold design to enhance cardiac repair.[136,147,158]

Additional engineering layers can further augment repair. Growth factor loading has been used to improve graft survival and vascularization in rodent and porcine MI models, for example through *IGF-1* mRNA-enriched patches or bound VEGF- and SDF-1-based approaches that recruit endogenous reparative cells and promote neovascularization after MI (Figure 4B).[141,159–161] Genome editing also allows implanted cells to be modified before transplantation. These strategies include engineering hypoimmunogenic "universal donor" cardiomyocytes to reduce graft rejection[138,162,163] and modifying electrophysiological properties to reduce automaticity and arrhythmia risk from grafted hiPSC-CMs (Figure 4C).[152]

OUTLOOK AND CONCLUSIONS

hiPSC-based MI models have evolved from simple hypoxia-reoxygenation assays in immature cardiomyocytes to multi-cell type 3D tissues and engineered microphysiological systems that capture features of ischemic gradients, scar formation, arrhythmias, and multicellular wound-healing responses. However, current models still capture selected windows of IHD pathophysiology and few integrate the vascular, immune, stromal, and neuronal components required to fully mimic the inflammatory and reparative processes that shape post-infarct outcome. Despite these limitations, this expanding toolkit helps address gaps left by animal models and reductionist human systems, enabling mechanistic and translational studies in settings that increasingly reflect key aspects of the human infarcted heart.

The next challenge is therefore not simply to increase model complexity but to develop fit-for-purpose systems aligned with the biological phase of MI being studied, including acute ischemic injury, IRI, inflammatory repair, fibrosis, and long-term remodeling. Incorporating vascular, immune, and stromal compartments, together with improved cardiomyocyte maturation, should bring hiPSC platforms closer to the biology of human infarct repair while retaining the robustness and scalability required for their intended CoU.

Successful translation will also depend on standardization, reproducibility, and regulatory confidence. Both the FDA and EMA support the use of scientifically validated NAMs, including cell-based systems, organ-on-chip models, and computational approaches, in medicine development and safety assessment.[120,164] Future hiPSC models of MI and IHD will therefore need to combine biological sophistication with robust performance, clear CoU, and clinically anchored readouts. For regenerative applications, translation will depend not only on efficacy but also on scalable GMP-compliant manufacturing, robust product characterization and potency testing, immune compatibility, and long-term safety. Reliable cryopreservation of large cell doses without viability or functional loss,[165] together with practical delivery logistics, will also be required before these therapies become clinically deployable and cost-effective.

A further priority is data integration. Multi-omics, high-content phenotyping, and machine-learning approaches can extract mechanistic and predictive information from hiPSC-based systems, while "clinical trials in a dish" aim to model variability

across genetically diverse populations. Integrating patient-specific hiPSC phenotypes, such as ischemia tolerance, fibrotic propensity, or proarrhythmic susceptibility, with multiscale computational models and cardiovascular digital twins could connect genotype and cellular behavior with organ-level function and clinical response. Together, improvements in model fidelity, context-specific benchmarking, and data integration should increase the predictive and translational value of hiPSC-based platforms for cardioprotection and repair.

ACKNOWLEDGMENTS

The authors thank their colleagues at the Leiden University Medical Center, Amy Lucassen, Marc Vila Cuenca, Nikola Popovic, and Loukia Yiangou, for their input on the text and figure design. This work was supported by the NWO Open Competition XS program (#OCENW.XS23.4.123), a Marie Sklodowska-Curie fellowship (MiniMI #101152209), and the Netherlands Organisation for Health Research and Development (ZonMW; PSIDER #10250022110004). The Novo Nordisk Foundation Center for Stem Cell Medicine (reNEW) is supported by the Novo Nordisk Foundation (NNF21CC0073729). Some image constructs were adapted from Servier Medical Art (https://smart.servier.com) licensed under CC BY 4.0. During preparation of this work, the authors used Perplexity-Pro to improve the readability and clarity of the text. The authors subsequently reviewed and edited the text as appropriate and take full responsibility for the content of the publication.

AUTHOR CONTRIBUTIONS

B.B.J., conceptualization, investigation, writing – original draft, and visualization; R.P.D. and C.L.M., writing – review & editing. All authors discussed and approved the manuscript.

DECLARATION OF INTERESTS

C.L.M. is a co-founder of Pluriomics B.V. (now Ncardia B.V.) and has advisory roles with HeartBeat.bio AG, Angios GmbH, Mogrify Limited, and Sartorius AG. R.P.D. declares research funding from Sartorius AG for an unrelated study.

SUPPLEMENTAL INFORMATION

Supplemental information can be found online at https://doi.org/10.1016/j.xcrm.2026.103079.

REFERENCES

1. Chong, B., Jayabaskaran, J., Jauhari, S.M., Chan, S.P., Goh, R., Kueh, M.T.W., Li, H., Chin, Y.H., Kong, G., Anand, V.V., et al. (2025). Global burden of cardiovascular diseases: projections from 2025 to 2050. Eur. J. Prev. Cardiol. *32*, 1001–1015. https://doi.org/10.1093/eurjpc/zwae281.
2. Lindstrom, M., Decleene, N., Dorsey, H., Fuster, V., Johnson, C.O., Legrand, K.E., Mensah, G.A., Razo, C., Stark, B., Varieur Turco, J., and Roth, G.A. (2022). Global Burden of Cardiovascular Diseases and Risks Collaboration, 1990-2021. J. Am. Coll. Cardiol. *80*, 2372–2425. https://doi.org/10.1016/j.jacc.2022.11.001.
3. Bays, H.E., Kulkarni, A., German, C., Satish, P., Iluyomade, A., Dudum, R., Thakkar, A., Rifai, M.A., Mehta, A., Thobani, A., et al. (2022). Ten things to know about ten cardiovascular disease risk factors - 2022. Am. J. Prev. Cardiol. *10*, 100342. https://doi.org/10.1016/j.ajpc.2022.100342.
4. Kazi, D.S., Elkind, M.S.V., Deutsch, A., Dowd, W.N., Heidenreich, P., Khavjou, O., Mark, D., Mussolino, M.E., Ovbiagele, B., Patel, S.S., et al. (2024). Forecasting the Economic Burden of Cardiovascular Disease and Stroke in the United States Through 2050: A Presidential Advisory From the American Heart Association. Circulation *150*, e89–e101. https://doi.org/10.1161/CIR.0000000000001258.
5. Luengo-Fernandez, R., Walli-Attaei, M., Gray, A., Torbica, A., Maggioni, A.P., Huculeci, R., Bairami, F., Aboyans, V., Timmis, A.D., Vardas, P., and Leal, J. (2023). Economic burden of cardiovascular diseases in the European Union: a population-based cost study. Eur. Heart J. *44*, 4752–4767. https://doi.org/10.1093/eurheartj/ehad583.
6. Leonard, E.A., and Marshall, R.J. (2018). Cardiovascular Disease in Women. Prim. Care *45*, 131–141. https://doi.org/10.1016/j.pop.2017.10.004.
7. Timmis, A., Vardas, P., Townsend, N., Torbica, A., Katus, H., De Smedt, D., Gale, C.P., Maggioni, A.P., Petersen, S.E., Huculeci, R., et al. (2022). European Society of Cardiology: cardiovascular disease statistics 2021: Executive Summary. Eur. Heart J. Qual. Care Clin. Outcomes *8*, 377–382. https://doi.org/10.1093/ehjqcco/qcac014.
8. Heusch, G. (2022). Coronary blood flow in heart failure: cause, consequence and bystander. Basic Res. Cardiol. *117*, 1. https://doi.org/10.1007/s00395-022-00909-8.
9. Heusch, G. (2024). Myocardial ischemia/reperfusion: Translational pathophysiology of ischemic heart disease. Med *5*, 10–31. https://doi.org/10.1016/j.medj.2023.12.007.
10. Townsend, N., Kazakiewicz, D., Lucy Wright, F., Timmis, A., Huculeci, R., Torbica, A., Gale, C.P., Achenbach, S., Weidinger, F., and Vardas, P. (2022). Epidemiology of cardiovascular disease in Europe. Nat. Rev. Cardiol. *19*, 133–143. https://doi.org/10.1038/s41569-021-00607-3.
11. Suman, S., Pravalika, J., Manjula, P., and Farooq, U. (2023). Gender and CVD- Does It Really Matters? Curr. Probl. Cardiol. *48*, 101604. https://doi.org/10.1016/j.cpcardiol.2023.101604.
12. Frangogiannis, N.G. (2015). Pathophysiology of Myocardial Infarction. Compr. Physiol. *5*, 1841–1875. https://doi.org/10.1002/cphy.c150006.
13. Mehta, P.K., Quesada, O., Al-Badri, A., Fleg, J.L., Volgman, A.S., Pepine, C.J., Merz, C.N.B., and Shaw, L.J. (2022). Ischemia and no obstructive coronary arteries in patients with stable ischemic heart disease. Int. J. Cardiol. *348*, 1–8. https://doi.org/10.1016/j.ijcard.2021.12.013.
14. Murry, C.E., Reinecke, H., and Pabon, L.M. (2006). Regeneration gaps: observations on stem cells and cardiac repair. J. Am. Coll. Cardiol. *47*, 1777–1785. https://doi.org/10.1016/j.jacc.2006.02.002.
15. Kastrati, A., Coughlan, J.J., and Ndrepepa, G. (2021). Primary PCI, Late Presenting STEMI, and the Limits of Time. J. Am. Coll. Cardiol. *78*, 1306–1308. https://doi.org/10.1016/j.jacc.2021.08.001.
16. Zhang, Q., Wang, L., Wang, S., Cheng, H., Xu, L., Pei, G., Wang, Y., Fu, C., Jiang, Y., He, C., and Wei, Q. (2022). Signaling pathways and targeted therapy for myocardial infarction. Signal Transduct. Target. Ther. *7*, 78. https://doi.org/10.1038/s41392-022-00925-z.
17. Schäfer, A., König, T., Bauersachs, J., and Akin, M. (2022). Novel Therapeutic Strategies to Reduce Reperfusion Injury After Acute Myocardial Infarction. Curr. Probl. Cardiol. *47*, 101398. https://doi.org/10.1016/j.cpcardiol.2022.101398.
18. Gao, Y., and Galis, Z.S. (2021). Exploring the Role of Endothelial Cell Resilience in Cardiovascular Health and Disease. Arterioscler. Thromb. Vasc. Biol. *41*, 179–185. https://doi.org/10.1161/ATVBAHA.120.314346.
19. Avolio, E., Campagnolo, P., Katare, R., and Madeddu, P. (2024). The role of cardiac pericytes in health and disease: therapeutic targets for myocardial infarction. Nat. Rev. Cardiol. *21*, 106–118. https://doi.org/10.1038/s41569-023-00913-y.
20. Cadosch, N., Gil-Cruz, C., Perez-Shibayama, C., and Ludewig, B. (2024). Cardiac Fibroblastic Niches in Homeostasis and Inflammation. Circ. Res. *134*, 1703–1717. https://doi.org/10.1161/CIRCRESAHA.124.323892.
21. Chistiakov, D.A., Melnichenko, A.A., Myasoedova, V.A., Grechko, A.V., and Orekhov, A.N. (2017). Mechanisms of foam cell formation in atherosclerosis. J. Mol. Med. *95*, 1153–1165. https://doi.org/10.1007/s00109-017-1575-8.

22. Libby, P. (2021). The changing landscape of atherosclerosis. Nature *592*, 524–533. https://doi.org/10.1038/s41586-021-03392-8.

23. Buja, L.M. (2023). Pathobiology of Myocardial Ischemia and Reperfusion Injury: Models, Modes, Molecular Mechanisms, Modulation, and Clinical Applications. Cardiol. Rev. *31*, 252–264. https://doi.org/10.1097/CRD.0000000000000440.

24. Bugger, H., and Pfeil, K. (2020). Mitochondrial ROS in myocardial ischemia reperfusion and remodeling. Biochim. Biophys. Acta. Mol. Basis Dis. *1866*, 165768. https://doi.org/10.1016/j.bbadis.2020.165768.

25. Davidson, S.M., Adameová, A., Barile, L., Cabrera-Fuentes, H.A., Lazou, A., Pagliaro, P., Stensløkken, K.O., and Garcia-Dorado, D.; EU-CARDIOPROTECTION COST Action CA16225 (2020). Mitochondrial and mitochondrial-independent pathways of myocardial cell death during ischaemia and reperfusion injury. J. Cell Mol. Med. *24*, 3795–3806. https://doi.org/10.1111/jcmm.15127.

26. Konstantinidis, K., Whelan, R.S., and Kitsis, R.N. (2012). Mechanisms of cell death in heart disease. Arterioscler. Thromb. Vasc. Biol. *32*, 1552–1562. https://doi.org/10.1161/ATVBAHA.111.224915.

27. Canty, J.M., Jr. (2022). Myocardial injury, troponin release, and cardiomyocyte death in brief ischemia, failure, and ventricular remodeling. Am. J. Physiol. Heart Circ. Physiol. *323*, 1–15. https://doi.org/10.1152/ajpheart.00093.2022.

28. Budhram-Mahadeo, V., Fujita, R., Bitsi, S., Sicard, P., and Heads, R. (2014). Co-expression of POU4F2/Brn-3b with p53 may be important for controlling expression of pro-apoptotic genes in cardiomyocytes following ischaemic/hypoxic insults. Cell Death Dis. *5*, e1503. https://doi.org/10.1038/cddis.2014.452.

29. Yap, J., Irei, J., Lozano-Gerona, J., Vanapruks, S., Bishop, T., and Boisvert, W.A. (2023). Macrophages in cardiac remodelling after myocardial infarction. Nat. Rev. Cardiol. *20*, 373–385. https://doi.org/10.1038/s41569-022-00823-5.

30. Feng, Q., Li, Q., Zhou, H., Sun, L., Lin, C., Jin, Y., Wang, D., and Guo, G. (2022). The role of major immune cells in myocardial infarction. Front. Immunol. *13*, 1084460. https://doi.org/10.3389/fimmu.2022.1084460.

31. Kawaguchi, M., Takahashi, M., Hata, T., Kashima, Y., Usui, F., Morimoto, H., Izawa, A., Takahashi, Y., Masumoto, J., Koyama, J., et al. (2011). Inflammasome activation of cardiac fibroblasts is essential for myocardial ischemia/reperfusion injury. Circulation *123*, 594–604. https://doi.org/10.1161/CIRCULATIONAHA.110.982777.

32. Frangogiannis, N.G. (2014). The inflammatory response in myocardial injury, repair, and remodelling. Nat. Rev. Cardiol. *11*, 255–265. https://doi.org/10.1038/nrcardio.2014.28.

33. Alex, L., and Frangogiannis, N.G. (2019). Pericytes in the infarcted heart. Vasc. Biol. *1*, H23–H31. https://doi.org/10.1530/VB-19-0007.

34. Talman, V., and Ruskoaho, H. (2016). Cardiac fibrosis in myocardial infarction-from repair and remodeling to regeneration. Cell Tissue Res. *365*, 563–581. https://doi.org/10.1007/s00441-016-2431-9.

35. Sanjaya, A. (2025). Transcriptomic dynamics of cardiac remodeling after myocardial infarction. Sci. Rep. *15*, 36720. https://doi.org/10.1038/s41598-025-20736-w.

36. Zhao, T., Zhao, W., Chen, Y., Ahokas, R.A., and Sun, Y. (2010). Vascular endothelial growth factor (VEGF)-A: role on cardiac angiogenesis following myocardial infarction. Microvasc. Res. *80*, 188–194. https://doi.org/10.1016/j.mvr.2010.03.014.

37. Sutton, M.G., and Sharpe, N. (2000). Left ventricular remodeling after myocardial infarction: pathophysiology and therapy. Circulation *101*, 2981–2988. https://doi.org/10.1161/01.cir.101.25.2981.

38. Mendonca Costa, C., Plank, G., Rinaldi, C.A., Niederer, S.A., and Bishop, M.J. (2018). Modeling the Electrophysiological Properties of the Infarct Border Zone. Front. Physiol. *9*, 356. https://doi.org/10.3389/fphys.2018.00356.

39. Triposkiadis, F., Briasoulis, A., Kitai, T., Magouliotis, D., Athanasiou, T., Skoularigis, J., and Xanthopoulos, A. (2024). The sympathetic nervous system in heart failure revisited. Heart Fail. Rev. *29*, 355–365. https://doi.org/10.1007/s10741-023-10345-y.

40. Frangogiannis, N.G. (2021). Cardiac fibrosis. Cardiovasc. Res. *117*, 1450–1488. https://doi.org/10.1093/cvr/cvaa324.

41. Lindsey, M.L., Bolli, R., Canty, J.M., Jr., Du, X.J., Frangogiannis, N.G., Frantz, S., Gourdie, R.G., Holmes, J.W., Jones, S.P., Kloner, R.A., et al. (2018). Guidelines for experimental models of myocardial ischemia and infarction. Am. J. Physiol. Heart Circ. Physiol. *314*, H812–H838. https://doi.org/10.1152/ajpheart.00335.2017.

42. Martin, T.P., Macdonald, E.A., Elbassioni, A.A.M., O'toole, D., Zaeri, A.A.I., Nicklin, S.A., Gray, G.A., and Loughrey, C.M. (2022). Preclinical models of myocardial infarction: from mechanism to translation. Br. J. Pharmacol. *179*, 770–791. https://doi.org/10.1111/bph.15595.

43. Shin, H.S., Shin, H.H., and Shudo, Y. (2021). Current Status and Limitations of Myocardial Infarction Large Animal Models in Cardiovascular Translational Research. Front. Bioeng. Biotechnol. *9*, 673683. https://doi.org/10.3389/fbioe.2021.673683.

44. Cesarovic, N., Lipiski, M., Falk, V., and Emmert, M.Y. (2020). Animals in cardiovascular research. Eur. Heart J. *41*, 200–203. https://doi.org/10.1093/eurheartj/ehz933.

45. De Villiers, C., and Riley, P.R. (2020). Mouse models of myocardial infarction: comparing permanent ligation and ischaemia-reperfusion. Dis. Model. Mech. *13*, dmm046565. https://doi.org/10.1242/dmm.046565.

46. Kaese, S., and Verheule, S. (2012). Cardiac electrophysiology in mice: a matter of size. Front. Physiol. *3*, 345. https://doi.org/10.3389/fphys.2012.00345.

47. Doevendans, P.A., Daemen, M.J., De Muinck, E.D., and Smits, J.F. (1998). Cardiovascular phenotyping in mice. Cardiovasc. Res. *39*, 34–49. https://doi.org/10.1016/s0008-6363(98)00073-x.

48. Krishnan, A., Samtani, R., Dhanantwari, P., Lee, E., Yamada, S., Shiota, K., Donofrio, M.T., Leatherbury, L., and Lo, C.W. (2014). A detailed comparison of mouse and human cardiac development. Pediatr. Res. *76*, 500–507. https://doi.org/10.1038/pr.2014.128.

49. Janssen, P.M.L., Biesiadecki, B.J., Ziolo, M.T., and Davis, J.P. (2016). The Need for Speed: Mice, Men, and Myocardial Kinetic Reserve. Circ. Res. *119*, 418–421. https://doi.org/10.1161/CIRCRESAHA.116.309126.

50. Arora, A., Nain, P., Kumari, R., and Kaur, J. (2021). Major Causes Associated with Clinical Trials Failure and Selective Strategies to Reduce these Consequences: A Review. Arch. Pharm. Pract. *12*, 45–53. https://doi.org/10.51847/yjqdk2wtgx.

51. Mummery, C.L., Zhang, J., Ng, E.S., Elliott, D.A., Elefanty, A.G., and Kamp, T.J. (2012). Differentiation of human embryonic stem cells and induced pluripotent stem cells to cardiomyocytes: a methods overview. Circ. Res. *111*, 344–358. https://doi.org/10.1161/CIRCRESAHA.110.227512.

52. Takahashi, K., Tanabe, K., Ohnuki, M., Narita, M., Ichisaka, T., Tomoda, K., and Yamanaka, S. (2007). Induction of pluripotent stem cells from adult human fibroblasts by defined factors. Cell *131*, 861–872. https://doi.org/10.1016/j.cell.2007.11.019.

53. Junying, Y., Maxim, A.V., Kim, S.-O., Jessica, A.-B., Jennifer, L.F., Shulan, T., Jeff, N., Gundrun, A.J., Victor, R., Ron, S., et al. (2007). Induced Pluripotent Stem Cell Lines Derived from Human Somatic Cells. Science *318*, 1917–1920. https://doi.org/10.1126/science.1151526.

54. Burridge, P.W., Holmström, A., and Wu, J.C. (2015). Chemically Defined Culture and Cardiomyocyte Differentiation of Human Pluripotent Stem Cells. Curr. Protoc. Hum. Genet. *87*, 21.3.1–21.3.15. https://doi.org/10.1002/0471142905.hg2103s87.

55. Bourque, K., Jones-Tabah, J., Pétrin, D., Martin, R.D., Tanny, J.C., and Hébert, T.E. (2023). Comparing the signaling and transcriptome profiling landscapes of human iPSC-derived and primary rat neonatal cardiomyocytes. Sci. Rep. *13*, 12248. https://doi.org/10.1038/s41598-023-39525-4.

56. Joukar, S. (2021). A comparative review on heart ion channels, action potentials and electrocardiogram in rodents and human: extrapolation of

experimental insights to clinic. Lab. Anim. Res. *37*, 25. https://doi.org/10.1186/s42826-021-00102-3.

57. Davis, R.P., Van Den Berg, C.W., Casini, S., Braam, S.R., and Mummery, C.L. (2011). Pluripotent stem cell models of cardiac disease and their implication for drug discovery and development. Trends Mol. Med. *17*, 475–484. https://doi.org/10.1016/j.molmed.2011.05.001.

58. Begovic, M., Schneider, L., Zhou, X., Hamdani, N., Akin, I., and El-Battrawy, I. (2024). The Role of Human-Induced Pluripotent Stem Cells in Studying Cardiac Channelopathies. Int. J. Mol. Sci. *25*, 12034. https://doi.org/10.3390/ijms252212034.

59. Liang, P., Sallam, K., Wu, H., Li, Y., Itzhaki, I., Garg, P., Zhang, Y., Vermglinchan, V., Lan, F., Gu, M., et al. (2016). Patient-Specific and Genome-Edited Induced Pluripotent Stem Cell-Derived Cardiomyocytes Elucidate Single-Cell Phenotype of Brugada Syndrome. J. Am. Coll. Cardiol. *68*, 2086–2096. https://doi.org/10.1016/j.jacc.2016.07.779.

60. Kim, C., Wong, J., Wen, J., Wang, S., Wang, C., Spiering, S., Kan, N.G., Forcales, S., Puri, P.L., Leone, T.C., et al. (2013). Studying arrhythmogenic right ventricular dysplasia with patient-specific iPSCs. Nature *494*, 105–110. https://doi.org/10.1038/nature11799.

61. Karakikes, I., Stillitano, F., Nonnenmacher, M., Tzimas, C., Sanoudou, D., Termglinchan, V., Kong, C.W., Rushing, S., Hansen, J., Ceholski, D., et al. (2015). Correction of human phospholamban R14del mutation associated with cardiomyopathy using targeted nucleases and combination therapy. Nat. Commun. *6*, 6955. https://doi.org/10.1038/ncomms7955.

62. Van Der Wilt, C.N., Veltrop, R.J.A., Janssens, M.H., Bakker, I., Stillitano, F., Sluijter, J.P.G., Van Laake, L.W., Van Der Velden, J., Villard, E., Montag, J., et al. (2026). Setting the stage for cardiomyopathy gene editing trials: a systematic review of isogenic pair use in human induced pluripotent stem cell-derived cardiomyocyte research. Eur. Heart J. Open *6*, oeaf161. https://doi.org/10.1093/ehjopen/oeaf161.

63. Smith, J.G.W., Owen, T., Bhagwan, J.R., Mosqueira, D., Scott, E., Mannhardt, I., Patel, A., Barriales-Villa, R., Monserrat, L., Hansen, A., et al. (2018). Isogenic Pairs of hiPSC-CMs with Hypertrophic Cardiomyopathy/LVNC-Associated ACTC1 E99K Mutation Unveil Differential Functional Deficits. Stem Cell Rep. *11*, 1226–1243. https://doi.org/10.1016/j.stemcr.2018.10.006.

64. Mosqueira, D., Mannhardt, I., Bhagwan, J.R., Lis-Slimak, K., Katili, P., Scott, E., Hassan, M., Prondzynski, M., Harmer, S.C., Tinker, A., et al. (2018). CRISPR/Cas9 editing in human pluripotent stem cell-cardiomyocytes highlights arrhythmias, hypocontractility, and energy depletion as potential therapeutic targets for hypertrophic cardiomyopathy. Eur. Heart J. *39*, 3879–3892. https://doi.org/10.1093/eurheartj/ehy249.

65. Hofbauer, P., Jahnel, S.M., Papai, N., Giesshammer, M., Deyett, A., Schmidt, C., Penc, M., Tavernini, K., Grdseloff, N., Meledeth, C., et al. (2021). Cardioids reveal self-organizing principles of human cardiogenesis. Cell *184*, 3299–3317.e22. https://doi.org/10.1016/j.cell.2021.04.034.

66. Meier, A.B., Zawada, D., De Angelis, M.T., Martens, L.D., Santamaria, G., Zengerle, S., Nowak-Imialek, M., Kornherr, J., Zhang, F., Tian, Q., et al. (2023). Epicardioid single-cell genomics uncovers principles of human epicardium biology in heart development and disease. Nat. Biotechnol. *41*, 1787–1800. https://doi.org/10.1038/s41587-023-01718-7.

67. Wu, P., Deng, G., Sai, X., Guo, H., Huang, H., and Zhu, P. (2021). Maturation strategies and limitations of induced pluripotent stem cell-derived cardiomyocytes. Biosci. Rep. *41*, BSR20200833. https://doi.org/10.1042/BSR20200833.

68. Sacchetto, C., Vitiello, L., De Windt, L.J., Rampazzo, A., and Calore, M. (2020). Modeling Cardiovascular Diseases with hiPSC-Derived Cardiomyocytes in 2D and 3D Cultures. Int. J. Mol. Sci. *21*, 3404. https://doi.org/10.3390/ijms21093404.

69. Li, W., Luo, X., Strano, A., Arun, S., Gamm, O., Poetsch, M.S., Hasse, M., Steiner, R.P., Fischer, K., Pöche, J., et al. (2025). Comprehensive promotion of iPSC-CM maturation by integrating metabolic medium with nanopatterning and electrostimulation. Nat. Commun. *16*, 2785. https://doi.org/10.1038/s41467-025-58044-6.

70. Zhu, K., Bao, X., Wang, Y., Lu, T., and Zhang, L. (2023). Human induced pluripotent stem cell (hiPSC)-derived cardiomyocyte modelling of cardiovascular diseases for natural compound discovery. Biomed. Pharmacother. *157*, 113970. https://doi.org/10.1016/j.biopha.2022.113970.

71. Joshi, J., Albers, C., Smole, N., Guo, S., and Smith, S.A. (2024). Human induced pluripotent stem cell-derived cardiomyocytes (iPSC-CMs) for modeling cardiac arrhythmias: strengths, challenges and potential solutions. Front. Physiol. *15*, 1475152. https://doi.org/10.3389/fphys.2024.1475152.

72. Häkli, M., Kreutzer, J., Mäki, A.J., Välimäki, H., Lappi, H., Huhtala, H., Kallio, P., Aalto-Setälä, K., and Pekkanen-Mattila, M. (2021). Human induced pluripotent stem cell-based platform for modeling cardiac ischemia. Sci. Rep. *11*, 4153. https://doi.org/10.1038/s41598-021-83740-w.

73. Hidalgo, A., Glass, N., Ovchinnikov, D., Yang, S.K., Zhang, X., Mazzone, S., Chen, C., Wolvetang, E., and Cooper-White, J. (2018). Modelling ischemia-reperfusion injury (IRI) in vitro using metabolically matured induced pluripotent stem cell-derived cardiomyocytes. APL Bioeng. *2*, 026102. https://doi.org/10.1063/1.5000746.

74. Davis, J., Chouman, A., Creech, J., Monteiro Da Rocha, A., Ponce-Balbuena, D., Jimenez Vazquez, E.N., Nichols, R., Lozhkin, A., Madamanchi, N.R., Campbell, K.F., and Herron, T.J. (2021). In vitro model of ischemic heart failure using human induced pluripotent stem cell-derived cardiomyocytes. JCI Insight *6*, e134368. https://doi.org/10.1172/jci.insight.134368.

75. Jensen, C., and Teng, Y. (2020). Is It Time to Start Transitioning From 2D to 3D Cell Culture? Front. Mol. Biosci. *7*, 33. https://doi.org/10.3389/fmolb.2020.00033.

76. Mannhardt, I., Breckwoldt, K., Letuffe-Brenière, D., Schaaf, S., Schulz, H., Neuber, C., Benzin, A., Werner, T., Eder, A., Schulze, T., et al. (2016). Human Engineered Heart Tissue: Analysis of Contractile Force. Stem Cell Rep. *7*, 29–42. https://doi.org/10.1016/j.stemcr.2016.04.011.

77. Polonchuk, L., Chabria, M., Badi, L., Hoflack, J.-C., Figtree, G., Davies, M.J., and Gentile, C. (2017). Cardiac spheroids as promising in vitro models to study the human heart microenvironment. Sci. Rep. *7*, 7005. https://doi.org/10.1038/s41598-017-06385-8.

78. Giacomelli, E., Meraviglia, V., Campostrini, G., Cochrane, A., Cao, X., Van Helden, R.W.J., Krotenberg Garcia, A., Mircea, M., Kostidis, S., Davis, R.P., et al. (2020). Human-iPSC-Derived Cardiac Stromal Cells Enhance Maturation in 3D Cardiac Microtissues and Reveal Non-cardiomyocyte Contributions to Heart Disease. Cell Stem Cell *26*, 862–879.e11. https://doi.org/10.1016/j.stem.2020.05.004.

79. Liu, B., Wang, S., Ma, H., Deng, Y., Du, J., Zhao, Y., and Chen, Y. (2025). Heart-on-a-chip: a revolutionary organ-on-chip platform for cardiovascular disease modeling. J. Transl. Med. *23*, 132. https://doi.org/10.1186/s12967-024-05986-y.

80. Peletier, M., Zhang, X., Klein, S., and Kroon, J. (2024). Multicellular 3D models to study myocardial ischemia-reperfusion injury. Front. Cell Dev. Biol. *12*, 1494911. https://doi.org/10.3389/fcell.2024.1494911.

81. Chen, T., and Vunjak-Novakovic, G. (2019). Human Tissue-Engineered Model of Myocardial Ischemia-Reperfusion Injury. Tissue Eng. Part A *25*, 711–724. https://doi.org/10.1089/ten.TEA.2018.0212.

82. Sebastião, M.J., Gomes-Alves, P., Reis, I., Sanchez, B., Palacios, I., Serra, M., and Alves, P.M. (2020). Bioreactor-based 3D human myocardial ischemia/reperfusion in vitro model: a novel tool to unveil key paracrine factors upon acute myocardial infarction. Transl. Res. *215*, 57–74. https://doi.org/10.1016/j.trsl.2019.09.001.

83. Acun, A., Nguyen, T.D., and Zorlutuna, P. (2019). In vitro aged, hiPSC-origin engineered heart tissue models with age-dependent functional deterioration to study myocardial infarction. Acta Biomater. *94*, 372–391. https://doi.org/10.1016/j.actbio.2019.05.064.

84. Yang, P., Zhu, L., Wang, S., Gong, J., Selvaraj, J.N., Ye, L., Chen, H., Zhang, Y., Wang, G., Song, W., et al. (2024). Engineered model of heart tissue repair for exploring fibrotic processes and therapeutic interventions. Nat. Commun. *15*, 7996. https://doi.org/10.1038/s41467-024-52221-9.

85. Paz-Artigas, L., González-Lana, S., Polo, N., Vicente, P., Montero-Calle, P., Martínez, M.A., Rábago, G., Serra, M., Prósper, F., Mazo, M.M., et al. (2024). Generation of Self-Induced Myocardial Ischemia in Large-Sized Cardiac Spheroids without Alteration of Environmental Conditions Recreates Fibrotic Remodeling and Tissue Stiffening Revealed by Constriction Assays. ACS Biomater. Sci. Eng. *10*, 987–997. https://doi.org/10.1021/acsbiomaterials.3c01302.

86. Sharma, P., Liu Chung Ming, C., Wang, X., Bienvenu, L.A., Beck, D., Figtree, G., Boyle, A., and Gentile, C. (2022). Biofabrication of advanced in vitro3D models to study ischaemic and doxorubicin-induced myocardial damage. Biofabrication *14*, 025003. https://doi.org/10.1088/1758-5090/ac47d8.

87. Richards, D.J., Li, Y., Kerr, C.M., Yao, J., Beeson, G.C., Coyle, R.C., Chen, X., Jia, J., Damon, B., Wilson, R., et al. (2020). Human cardiac organoids for the modelling of myocardial infarction and drug cardiotoxicity. Nat. Biomed. Eng. *4*, 446–462. https://doi.org/10.1038/s41551-020-0539-4.

88. De Korte, T., Johnson, B.B., Kosmidis, G., Samson-Couterie, B., Mol, M.P.H., Van Helden, R.W.J., Razaghi, E., François, L., Meraviglia, V., Yiangou, L., et al. (2026). Industrialization of 3D hiPSC-cardiac microtissues for high-throughput cardiac safety and drug discovery screening. Trends Biotechnol. *44*, 1100–1127. https://doi.org/10.1016/j.tibtech.2025.11.016.

89. Ergir, E., Oliver-De La Cruz, J., Fernandes, S., Cassani, M., Niro, F., Pereira-Sousa, D., Vrbský, J., Vinarský, V., Perestrelo, A.R., Debellis, D., et al. (2022). Generation and maturation of human iPSC-derived 3D organotypic cardiac microtissues in long-term culture. Sci. Rep. *12*, 17409. https://doi.org/10.1038/s41598-022-22225-w.

90. Song, M., Choi, D.B., Im, J.S., Song, Y.N., Kim, J.H., Lee, H., An, J., Kim, A., Choi, H., Kim, J.C., et al. (2024). Modeling acute myocardial infarction and cardiac fibrosis using human induced pluripotent stem cell-derived multi-cellular heart organoids. Cell Death Dis. *15*, 308. https://doi.org/10.1038/s41419-024-06703-9.

91. Yadid, M., Lind, J.U., Ardoña, H.A.M., Sheehy, S.P., Dickinson, L.E., Eweje, F., Bastings, M.M.C., Pope, B., O'connor, B.B., Straubhaar, J.R., et al. (2020). Endothelial extracellular vesicles contain protective proteins and rescue ischemia-reperfusion injury in a human heart-on-chip. Sci. Transl. Med. *12*, eaax8005. https://doi.org/10.1126/scitranslmed.aax8005.

92. Nunes, S.S., Miklas, J.W., Liu, J., Aschar-Sobbi, R., Xiao, Y., Zhang, B., Jiang, J., Massé, S., Gagliardi, M., Hsieh, A., et al. (2013). Biowire: a platform for maturation of human pluripotent stem cell-derived cardiomyocytes. Nat. Methods *10*, 781–787. https://doi.org/10.1038/nmeth.2524.

93. Mastikhina, O., Moon, B.U., Williams, K., Hatkar, R., Gustafson, D., Mourad, O., Sun, X., Koo, M., Lam, A.Y.L., Sun, Y., et al. (2020). Human cardiac fibrosis-on-a-chip model recapitulates disease hallmarks and can serve as a platform for drug testing. Biomaterials *233*, 119741. https://doi.org/10.1016/j.biomaterials.2019.119741.

94. Wang, E.Y., Rafatian, N., Zhao, Y., Lee, A., Lai, B.F.L., Lu, R.X., Jekic, D., Davenport Huyer, L., Knee-Walden, E.J., Bhattacharya, S., et al. (2019). Biowire Model of Interstitial and Focal Cardiac Fibrosis. ACS Cent. Sci. *5*, 1146–1158. https://doi.org/10.1021/acscentsci.9b00052.

95. Low, L.A., Mummery, C., Berridge, B.R., Austin, C.P., and Tagle, D.A. (2021). Organs-on-chips: into the next decade. Nat. Rev. Drug Discov. *20*, 345–361. https://doi.org/10.1038/s41573-020-0079-3.

96. Ellis, B.W., Ronan, G., Ren, X., Bahcecioglu, G., Senapati, S., Anderson, D., Handberg, E., March, K.L., Chang, H.C., and Zorlutuna, P. (2022). Human Heart Anoxia and Reperfusion Tissue (HEART) Model for the Rapid Study of Exosome Bound miRNA Expression As Biomarkers for Myocardial Infarction. Small *18*, e2201330. https://doi.org/10.1002/smll.202201330.

97. Veldhuizen, J., Chavan, R., Moghadas, B., Park, J.G., Kodibagkar, V.D., Migrino, R.Q., and Nikkhah, M. (2022). Cardiac ischemia on-a-chip to investigate cellular and molecular response of myocardial tissue under hypoxia. Biomaterials *281*, 121336. https://doi.org/10.1016/j.biomaterials.2021.121336.

98. Ji, J., Tristan, M., Ketchum, F., Kuang, W., Fu, G., Ren, X., and Zorlutuna, P. (2025). Immune-integrated cardiac fibrosis-on-a-chip: a 3D microfluidic device for region-specific immune-cardiac crosstalk in the fibrotic heart. Lab Chip *25*, 6401–6414. https://doi.org/10.1039/d5lc00469a.

99. Thygesen, K., Alpert, J.S., Jaffe, A.S., Chaitman, B.R., Bax, J.J., Morrow, D.A., and White, H.D.; Executive Group on behalf of the Joint European Society of Cardiology ESC/American College of Cardiology ACC/American Heart Association AHA/World Heart Federation WHF Task Force for the Universal Definition of Myocardial Infarction (2018). Fourth Universal Definition of Myocardial Infarction (2018). J. Am. Coll. Cardiol. *72*, 2231–2264. https://doi.org/10.1016/j.jacc.2018.08.1038.

100. Kuppe, C., Ramirez Flores, R.O., Li, Z., Hayat, S., Levinson, R.T., Liao, X., Hannani, M.T., Tanevski, J., Wünnemann, F., Nagai, J.S., et al. (2022). Spatial multi-omic map of human myocardial infarction. Nature *608*, 766–777. https://doi.org/10.1038/s41586-022-05060-x.

101. Krysko, D.V., Vanden Berghe, T., D'herde, K., and Vandenabeele, P. (2008). Apoptosis and necrosis: detection, discrimination and phagocytosis. Methods *44*, 205–221. https://doi.org/10.1016/j.ymeth.2007.12.001.

102. Ma, R., Lu, D., Yang, Z., Ji, X., Tian, R., Xu, F., Chen, Y., and Li, C. (2025). Emerging therapy strategies for energy metabolism in acute myocardial infarction. J. Transl. Med. *23*, 1140. https://doi.org/10.1186/s12967-025-07088-9.

103. Pendergrass, W., Wolf, N., and Poot, M. (2004). Efficacy of MitoTracker Green and CMXrosamine to measure changes in mitochondrial membrane potentials in living cells and tissues. Cytometry. A. *61*, 162–169. https://doi.org/10.1002/cyto.a.20033.

104. Kitai, T., Kohsaka, S., Kato, T., Kato, E., Sato, K., Teramoto, K., Yaku, H., Akiyama, E., Ando, M., Izumi, C., et al. (2025). JCS/JHFS 2025 Guideline on Diagnosis and Treatment of Heart Failure. Circ. J. *89*, 1579–1581. https://doi.org/10.1253/circj.CJ-66-0242.

105. Gaballah, M., Penttinen, K., Kreutzer, J., Mäki, A.J., Kallio, P., and Aalto-Setälä, K. (2022). Cardiac Ischemia On-a-Chip: Antiarrhythmic Effect of Levosimendan on Ischemic Human-Induced Pluripotent Stem Cell-Derived Cardiomyocytes. Cells *11*, 1045. https://doi.org/10.3390/cells11061045.

106. Sala, L., Van Meer, B.J., Tertoolen, L.G.J., Bakkers, J., Bellin, M., Davis, R.P., Denning, C., Dieben, M.a.E., Eschenhagen, T., Giacomelli, E., et al. (2018). MUSCLEMOTION: A Versatile Open Software Tool to Quantify Cardiomyocyte and Cardiac Muscle Contraction In Vitro and In Vivo. Circ. Res. *122*, e5–e16. https://doi.org/10.1161/CIRCRESAHA.117.312067.

107. Lyra-Leite, D.M., Gutiérrez-Gutiérrez, Ó., Wang, M., Zhou, Y., Cyganek, L., and Burridge, P.W. (2022). A review of protocols for human iPSC culture, cardiac differentiation, subtype-specification, maturation, and direct reprogramming. STAR Protoc. *3*, 101560. https://doi.org/10.1016/j.xpro.2022.101560.

108. Zhang, T., Fan, L., Yao, F., Li, G., Lin, G., Yang, Y., Qian, F., Liu, C., Tang, Q., Wang, W., et al. (2026). Human PSC-derived sinoatrial node-cardiac plexus assembloids model innervation-associated maturation of pacemaker systems. Cell Stem Cell *33*, 945–963.e11. https://doi.org/10.1016/j.stem.2026.04.018.

109. Schmidt, C., Deyett, A., Ilmer, T., Haendeler, S., Torres Caballero, A., Novatchkova, M., Netzer, M.A., Ceci Ginistrelli, L., Mancheno Juncosa, E., Bhattacharya, T., et al. (2023). Multi-chamber cardioids unravel human heart development and cardiac defects. Cell *186*, 5587–5605.e27. https://doi.org/10.1016/j.cell.2023.10.030.

110. Schulze, M.L., Lemoine, M.D., Fischer, A.W., Scherschel, K., David, R., Riecken, K., Hansen, A., Eschenhagen, T., and Ulmer, B.M. (2019). Dissecting hiPSC-CM pacemaker function in a cardiac organoid model. Biomaterials *206*, 133–145. https://doi.org/10.1016/j.biomaterials.2019.03.023.

111. Rockel, A.F., Brunnbauer, T., Wagner, N., Gerull, B., Ergün, S., and Wörsdörfer, P. (2025). Tissue-resident macrophages co-develop with myocardial tissue in human induced pluripotent stem cell-derived organoids. Front. Cell Dev. Biol. *13*, 1629988. https://doi.org/10.3389/fcell.2025.1629988.

112. O'hern, C., Caywood, S., Aminova, S., Kiselev, A., Volmert, B., Cao, W., Wang, F., Dionise, M., Sewavi, M.L., Skoric, M., et al. (2025). Human heart-macrophage assembloids mimic immune-cardiac interactions and enable arrhythmia disease modeling. Cell Stem Cell *32*, 1671–1690.e13. https://doi.org/10.1016/j.stem.2025.09.011.

113. Patsy, M., Ge, K., Khodabukus, A., and Bursac, N. (2025). Development and modeling of cardiac autonomic innervation. Nat. Cardiovasc. Res. *4*, 1598–1615. https://doi.org/10.1038/s44161-025-00746-7.

114. Arslan, U., Brescia, M., Meraviglia, V., Nahon, D.M., Van Helden, R.W.J., Stein, J.M., Van Den Hil, F.E., Van Meer, B.J., Vila Cuenca, M., Mummery, C.L., and Orlova, V.V. (2023). Vascularized hiPSC-derived 3D cardiac microtissue on chip. Stem Cell Rep. *18*, 1394–1404. https://doi.org/10.1016/j.stemcr.2023.06.001.

115. Landau, S., Zhao, Y., Hamidzada, H., Kent, G.M., Okhovatian, S., Lu, R.X.Z., Liu, C., Wagner, K.T., Cheung, K., Shawky, S.A., et al. (2024). Primitive macrophages enable long-term vascularization of human heart-on-a-chip platforms. Cell Stem Cell *31*, 1222–1238.e10. https://doi.org/10.1016/j.stem.2024.05.011.

116. Brown, D.A., Maclellan, W.R., Laks, H., Dunn, J.C.Y., Wu, B.M., and Beygui, R.E. (2007). Analysis of oxygen transport in a diffusion-limited model of engineered heart tissue. Biotechnol. Bioeng. *97*, 962–975. https://doi.org/10.1002/bit.21295.

117. Rajakulasingam, R., Ferreira, P.F., Scott, A.D., Khalique, Z., Azzu, A., Molto, M., Conway, M., Falaschetti, E., Cheng, K., Hammersley, D.J., et al. (2025). Characterization of dynamic changes in cardiac microstructure after reperfused ST-elevation myocardial infarction by biphasic diffusion tensor cardiovascular magnetic resonance. Eur. Heart J. *46*, 454–469. https://doi.org/10.1093/eurheartj/ehae667.

118. Wang, H., Brown, P.C., Chow, E.C.Y., Ewart, L., Ferguson, S.S., Fitzpatrick, S., Freedman, B.S., Guo, G.L., Hedrich, W., Heyward, S., et al. (2021). 3D cell culture models: Drug pharmacokinetics, safety assessment, and regulatory consideration. Clin. Transl. Sci. *14*, 1659–1680. https://doi.org/10.1111/cts.13066.

119. Zushin, P.J.H., Mukherjee, S., and Wu, J.C. (2023). FDA Modernization Act 2.0: transitioning beyond animal models with human cells, organoids, and AI/ML-based approaches. J. Clin. Investig. *133*, e175824. https://doi.org/10.1172/JCI175824.

120. Gerke, S., Balamut, J., and Wagner, J.K. (2026). The FDA's plan to phase out animal testing. Trends Biotechnol. *44*, 2178–2181. https://doi.org/10.1016/j.tibtech.2025.12.011.

121. Tani, H., and Tohyama, S. (2022). Human Engineered Heart Tissue Models for Disease Modeling and Drug Discovery. Front. Cell Dev. Biol. *10*, 855763. https://doi.org/10.3389/fcell.2022.855763.

122. De Korte, T., Katili, P.A., Mohd Yusof, N.a.N., Van Meer, B.J., Saleem, U., Burton, F.L., Smith, G.L., Clements, P., Mummery, C.L., Eschenhagen, T., et al. (2020). Unlocking Personalized Biomedicine and Drug Discovery with Human Induced Pluripotent Stem Cell-Derived Cardiomyocytes: Fit for Purpose or Forever Elusive? Annu. Rev. Pharmacol. Toxicol. *60*, 529–551. https://doi.org/10.1146/annurev-pharmtox-010919-023309.

123. Gintant, G., Burridge, P., Gepstein, L., Harding, S., Herron, T., Hong, C., Jalife, J., and Wu, J.C. (2019). Use of Human Induced Pluripotent Stem Cell-Derived Cardiomyocytes in Preclinical Cancer Drug Cardiotoxicity Testing: A Scientific Statement From the American Heart Association. Circ. Res. *125*, e75–e92. https://doi.org/10.1161/RES.0000000000000291.

124. Colatsky, T., Fermini, B., Gintant, G., Pierson, J.B., Sager, P., Sekino, Y., Strauss, D.G., and Stockbridge, N. (2016). The Comprehensive in Vitro Proarrhythmia Assay (CiPA) initiative - Update on progress. J. Pharmacol. Toxicol. Methods *81*, 15–20. https://doi.org/10.1016/j.vascn.2016.06.002.

125. Dostanić, M., Wiendels, M., Windt, L.M., Mol, M.P.H., Van Den Hil, F.E., Davis, R.P., Orlova, V., Van Meer, B.J., Mastrangeli, M., and Mummery, C.L. (2025). Tapered Pillar Design for High-Precision Force Readout in Miniaturized Engineered Heart Tissues From Human Pluripotent Stem Cells. Adv. Healthc. Mater. *14*, e01664. https://doi.org/10.1002/adhm.202501664.

126. Raniga, K., Nasir, A., Vo, N.T.N., Vaidyanathan, R., Dickerson, S., Hilcove, S., Mosqueira, D., Mirams, G.R., Clements, P., Hicks, R., et al. (2024). Strengthening cardiac therapy pipelines using human pluripotent stem cell-derived cardiomyocytes. Cell Stem Cell *31*, 292–311. https://doi.org/10.1016/j.stem.2024.01.007.

127. Wu, X., Swanson, K., Yildirim, Z., Liu, W., Liao, R., and Wu, J.C. (2024). Clinical trials in-a-dish for cardiovascular medicine. Eur. Heart J. *45*, 4275–4290. https://doi.org/10.1093/eurheartj/ehae519.

128. Gaballah, M., Walls, K., Zakzook, F., Kreutzer, J., Levijoki, J., and Aalto-Setälä, K. (2025). A hypoxia-on-a-chip platform for modeling ischemic arrhythmogenesis and evaluating the effects of levosimendan and OR-1896 on ischemic human iPSC-derived cardiomyocytes. Front. Bioeng. Biotechnol. *13*, 1671013. https://doi.org/10.3389/fbioe.2025.1671013.

129. Zhang, H., Thai, P.N., Shivnaraine, R.V., Ren, L., Wu, X., Siepe, D.H., Liu, Y., Tu, C., Shin, H.S., Caudal, A., et al. (2024). Multiscale drug screening for cardiac fibrosis identifies MD2 as a therapeutic target. Cell *187*, 7143–7163.e22. https://doi.org/10.1016/j.cell.2024.09.034.

130. Burridge, P.W., Li, Y.F., Matsa, E., Wu, H., Ong, S.G., Sharma, A., Holmström, A., Chang, A.C., Coronado, M.J., Ebert, A.D., et al. (2016). Human induced pluripotent stem cell-derived cardiomyocytes recapitulate the predilection of breast cancer patients to doxorubicin-induced cardiotoxicity. Nat. Med. *22*, 547–556. https://doi.org/10.1038/nm.4087.

131. Kitani, T., Ong, S.G., Lam, C.K., Rhee, J.W., Zhang, J.Z., Oikonomopoulos, A., Ma, N., Tian, L., Lee, J., Telli, M.L., et al. (2019). Human-Induced Pluripotent Stem Cell Model of Trastuzumab-Induced Cardiac Dysfunction in Patients With Breast Cancer. Circulation *139*, 2451–2465. https://doi.org/10.1161/CIRCULATIONAHA.118.037357.

132. Matsa, E., Burridge, P.W., Yu, K.H., Ahrens, J.H., Termglinchan, V., Wu, H., Liu, C., Shukla, P., Sayed, N., Churko, J.M., et al. (2016). Transcriptome Profiling of Patient-Specific Human iPSC-Cardiomyocytes Predicts Individual Drug Safety and Efficacy Responses In Vitro. Cell Stem Cell *19*, 311–325. https://doi.org/10.1016/j.stem.2016.07.006.

133. Thangaraj, P.M., Benson, S.H., Oikonomou, E.K., Asselbergs, F.W., and Khera, R. (2024). Cardiovascular care with digital twin technology in the era of generative artificial intelligence. Eur. Heart J. *45*, 4808–4821. https://doi.org/10.1093/eurheartj/ehae619.

134. Givens, S.E., Andebrhan, A.A., Schmuck, E.G., Renaud, A., Xie, A., Ebrahimi-Barough, S., Abrahante, J.E., Stanis, N., Dudley, S., Dutton, J.R., and Ogle, B.M. (2025). Healthy human induced pluripotent stem cell-derived cardiomyocytes exhibit sex dimorphism even without the addition of hormones. Stem Cell. *43*, sxaf038. https://doi.org/10.1093/stmcls/sxaf038.

135. Martin, T.G., and Leinwand, L.A. (2024). Hearts apart: sex differences in cardiac remodeling in health and disease. J. Clin. Investig. *134*, e180074. https://doi.org/10.1172/JCI180074.

136. Bain, J.D., Barrs, R.W., and Mei, Y. (2026). Progress and challenges in transplantation of human pluripotent stem cell derived cardiomyocytes for cardiac therapy. NPJ Biomed. Innov. *3*, 2. https://doi.org/10.1038/s44385-025-00048-4.

137. Winkelbauer, M., Generali, M., Cheng, K., Emmert, M.Y., and Chansoria, P. (2026). Epicardial patches for myocardial repair: circumventing barriers to heal ischemic heart tissue. Trends Biotechnol. *0*, S0167–S7799.

138. Dulak, J., Zieliński, T., Józkowicz, A., and Łoboda, A. (2025). Pluripotent stem cell-based approaches for heart repair and the potential of genetic modifications. Mol. Ther. *33*, 4766–4783. https://doi.org/10.1016/j.ymthe.2025.07.009.

139. Li, J., Hu, S., Zhu, D., Huang, K., Mei, X., López de Juan Abad, B., and Cheng, K. (2021). All Roads Lead to Rome (the Heart): Cell Retention

CellPress
OPEN ACCESS

and Outcomes From Various Delivery Routes of Cell Therapy Products to the Heart. J. Am. Heart Assoc. *10*, e020402. https://doi.org/10.1161/JAHA.120.020402.

140. Montague, E.C., Ozcan, B., Sefton, E., Wulkan, F., Alibhai, F.J., and Laflamme, M.A. (2025). Human pluripotent stem cell-based cardiac repair: Lessons learned and challenges ahead. Adv. Drug Deliv. Rev. *222*, 115594. https://doi.org/10.1016/j.addr.2025.115594.

141. Ye, L., Chang, Y.H., Xiong, Q., Zhang, P., Zhang, L., Somasundaram, P., Lepley, M., Swingen, C., Su, L., Wendel, J.S., et al. (2014). Cardiac repair in a porcine model of acute myocardial infarction with human induced pluripotent stem cell-derived cardiovascular cells. Cell Stem Cell *15*, 750–761. https://doi.org/10.1016/j.stem.2014.11.009.

142. Mattapally, S., Zhu, W., Fast, V.G., Gao, L., Worley, C., Kannappan, R., Borovjagin, A.V., and Zhang, J. (2018). Spheroids of cardiomyocytes derived from human-induced pluripotent stem cells improve recovery from myocardial injury in mice. Am. J. Physiol. Heart Circ. Physiol. *315*, H327–H339. https://doi.org/10.1152/ajpheart.00688.2017.

143. Tabei, R., Kawaguchi, S., Kanazawa, H., Tohyama, S., Hirano, A., Handa, N., Hishikawa, S., Teratani, T., Kunita, S., Fukuda, J., et al. (2019). Development of a transplant injection device for optimal distribution and retention of human induced pluripotent stem cell−derived cardiomyocytes. J. Heart Lung Transplant. *38*, 203–214. https://doi.org/10.1016/j.healun.2018.11.002.

144. Demkes, E., Cervera-Barea, A., Ebner-Peking, P., Wolf, M., Hochmann, S., Scheren, A.S., Bijsterveld, M., Van Oostveen, C.M., Jansen, M., Visser, J., et al. (2025). Human Cardiac Microtissues Display Improved Engraftment and Survival in a Porcine Model of Myocardial Infarction. J. Cardiovasc. Transl. Res. *18*, 512–528. https://doi.org/10.1007/s12265-025-10596-0.

145. Min, S., Kim, S., Sim, W.S., Choi, Y.S., Joo, H., Park, J.H., Lee, S.J., Kim, H., Lee, M.J., Jeong, I., et al. (2024). Versatile human cardiac tissues engineered with perfusable heart extracellular microenvironment for biomedical applications. Nat. Commun. *15*, 2564. https://doi.org/10.1038/s41467-024-46928-y.

146. Miyagawa, S., Kainuma, S., Kawamura, T., Suzuki, K., Ito, Y., Iseoka, H., Ito, E., Takeda, M., Sasai, M., Mochizuki-Oda, N., et al. (2022). Case report: Transplantation of human induced pluripotent stem cell-derived cardiomyocyte patches for ischemic cardiomyopathy. Front. Cardiovasc. Med. *9*, 950829. https://doi.org/10.3389/fcvm.2022.950829.

147. Jebran, A.F., Seidler, T., Tiburcy, M., Daskalaki, M., Kutschka, I., Fujita, B., Ensminger, S., Bremmer, F., Moussavi, A., Yang, H., et al. (2025). Engineered heart muscle allografts for heart repair in primates and humans. Nature *639*, 503–511. https://doi.org/10.1038/s41586-024-08463-0.

148. Kirkeby, A., Main, H., and Carpenter, M. (2025). Pluripotent stem-cell-derived therapies in clinical trial: A 2025 update. Cell Stem Cell *32*, 329–331. https://doi.org/10.1016/j.stem.2025.01.003.

149. Guan, X., Zhang, P., Wang, Q., Zhang, Y., Zhang, Y., Zhang, H., Zhang, H., Jiang, W., Wu, Y., Wang, X., et al. (2025). Human induced pluripotent stem cell-derived cardiomyocytes improve recovery from myocardial infarction in non-human primates. Stem Cell Res. Ther. *16*, 581. https://doi.org/10.1186/s13287-025-04664-0.

150. Zimmermann, W.H., Ensminger, S., Kutschka, I., Paitazoglou, C., Seidler, T., Brandenburg, S., Anker, S.D., Bader, N., Bergau, L., Bremmer, F., et al. (2026). Stem-Cell-Derived Biologic Ventricular Assist Tissue in Heart Failure. N. Engl. J. Med. *394*, 1991–2001. https://doi.org/10.1056/NEJMoa2513525.

151. Fan, L., Tang, Q., Wang, Y., Sun, H., Li, G., Yang, Y., Zhu, H., Liu, Z., Wang, H., Wang, H., et al. (2025). Injury-induced Clusterin+ cardiomyocytes suppress inflammation and promote regeneration in neonatal and adult hearts by reprogramming macrophages. Cell Stem Cell *32*, 1849–1868.e15. https://doi.org/10.1016/j.stem.2025.10.008.

152. Marchiano, S., Nakamura, K., Reinecke, H., Neidig, L., Lai, M., Kadota, S., Perbellini, F., Yang, X., Klaiman, J.M., Blakely, L.P., et al. (2023). Gene editing to prevent ventricular arrhythmias associated with cardiomyocyte cell therapy. Cell Stem Cell *30*, 741. https://doi.org/10.1016/j.stem.2023.04.010.

153. Wulkan, F., Romagnuolo, R., Qiang, B., Valdman Sadikov, T., Kim, K.P., Quesnel, E., Jiang, W., Andharia, N., Weyers, J.J., Ghugre, N.R., et al. (2024). Stem cell-derived cardiomyocytes expressing a dominant negative pacemaker HCN4 channel do not reduce the risk of graft-related arrhythmias. Front. Cardiovasc. Med. *11*, 1374881. https://doi.org/10.3389/fcvm.2024.1374881.

154. Saravanan, S., Sareen, N., Abu-El-Rub, E., Ashour, H., Sequiera, G.L., Ammar, H.I., Gopinath, V., Shamaa, A.A., Sayed, S.S.E., Moudgil, M., et al. (2018). Graphene Oxide-Gold Nanosheets Containing Chitosan Scaffold Improves Ventricular Contractility and Function After Implantation into Infarcted Heart. Sci. Rep. *8*, 15069. https://doi.org/10.1038/s41598-018-33144-0.

155. Karapehlivan, S.S., Danisik, M.N., Akdag, Z., Yildiz, E.N., Okoro, O.V., Nie, L., Shavandi, A., Ulag, S., Sahin, A., Dumludag, F., and Gunduz, O. (2023). Fabrication and In Vitro Characterization of Polycaprolactone/Graphene Oxide/Collagen Nanofibers for Myocardial Repair. Macromol. Mater. Eng. *309*, 2300189. https://doi.org/10.1002/mame.202300189.

156. Jang, J., Park, H.J., Kim, S.W., Kim, H., Park, J.Y., Na, S.J., Kim, H.J., Park, M.N., Choi, S.H., Park, S.H., et al. (2017). 3D printed complex tissue construct using stem cell-laden decellularized extracellular matrix bioinks for cardiac repair. Biomaterials *112*, 264–274. https://doi.org/10.1016/j.biomaterials.2016.10.026.

157. Bejleri, D., and Davis, M.E. (2019). Decellularized Extracellular Matrix Materials for Cardiac Repair and Regeneration. Adv. Healthc. Mater. *8*, e1801217. https://doi.org/10.1002/adhm.201801217.

158. Sridharan, D., Pervaiz, S., Nair, N.C., Mergaye, M.M., Islam, H., Blackstone, B.N., Ashraf, S.A., Alvi, S.B., Joseph, M., Varghese, J., et al. (2026). Safety and Engraftment of Aligned Cardiac Patches Loaded with hiPSC-CMs in a Large Animal Model of Myocardial Infarction. Theranostics *16*, 3978–3995. https://doi.org/10.7150/thno.121105.

159. Yan, B., Ai, X., Wang, H., Tan, Y., Gong, Y., Yang, L., Chen, Y., Lu, T., Liu, M., Luo, R., et al. (2026). Tissue-engineered cardiac patches enriched with IGF1 modified mRNA alleviate myocardial infarction by enhancing cell survival and angiogenesis. Mater. Today Bio *36*, 102686. https://doi.org/10.1016/j.mtbio.2025.102686.

160. Mei, X., and Cheng, K. (2020). Recent Development in Therapeutic Cardiac Patches. Front. Cardiovasc. Med. *7*, 610364. https://doi.org/10.3389/fcvm.2020.610364.

161. Sun, J., Zhao, Y., Li, Q., Chen, B., Hou, X., Xiao, Z., and Dai, J. (2016). Controlled Release of Collagen-Binding SDF-1alpha Improves Cardiac Function after Myocardial Infarction by Recruiting Endogenous Stem Cells. Sci. Rep. *6*, 26683. https://doi.org/10.1038/srep26683.

162. Deuse, T., Hu, X., Gravina, A., Wang, D., Tediashvili, G., De, C., Thayer, W.O., Wahl, A., Garcia, J.V., Reichenspurner, H., et al. (2019). Hypoimmunogenic derivatives of induced pluripotent stem cells evade immune rejection in fully immunocompetent allogeneic recipients. Nat. Biotechnol. *37*, 252–258. https://doi.org/10.1038/s41587-019-0016-3.

163. Silver, S.E., Howells, A.R., Arhontoulis, D.C., Randolph, L.N., Hyams, N.A., Barrs, R.W., Li, M., Kerr, C.M., Robino, R.A., Morningstar, J.E., et al. (2025). Hypoimmunogenic hPSC-derived cardiac organoids for immune evasion and heart repair. Preprint at bioRxiv. https://doi.org/10.1101/2025.04.09.648007.

164. Empl, M.T., Paul, P., Moriarty, O., Ponzano, S., Beken, S., Adler-Flindt, S., Theunissen, P., Andersen, K.J., Balks, E., Van Meer, P., et al. (2026). 3Rs at the European Medicines Agency: Past and future activities. NAMTA J. *2*, 100086. https://doi.org/10.1016/j.namjnl.2026.100086.

165. Massie, I., Selden, C., Hodgson, H., Fuller, B., Gibbons, S., and Morris, G.J. (2014). GMP cryopreservation of large volumes of cells for regenerative medicine: active control of the freezing process. Tissue Eng. Part C Methods *20*, 693–702. https://doi.org/10.1089/ten.TEC.2013.0571.

Cell Reports Medicine, Volume *7*

Supplemental information

Human iPSC models of myocardial infarction: From injury to repair and therapeutic discovery

Benjamin B. Johnson, Christine L. Mummery, and Richard P. Davis

Supplementary Table 1. Comparison of key strengths and limitations of hiPSC-based cardiac models and animal models in cardiovascular research

Feature	hiPSC-based cardiac models	Animal models
**Species relevance**	Human genetic background and human-relevant expression patterns	Provide intact physiology, but species differences can limit translation
**Patient specificity**	Can capture patient- or genotype-specific phenotypes	Limited patient specificity
**Ethics**	Avoid many animal-use issues, but still require donor consent and ethical oversight	Subject to animal ethics regulations and welfare constraints
**Scalability**	Expandable and compatible with medium- to high-throughput workflows	Lower throughput and constrained by cost, logistics, and animal use
**Maturity**	Often developmentally immature and may require maturation strategies	Native adult tissue context, although experimental animals may not reflect typical patient age or comorbidity
**Biological complexity**	Controlled and modular, but lack complete systemic, vascular, immune, and neurohumoral context	Capture integrated organ- and organism-level physiology
**Genetic manipulation**	Amenable to targeted engineering and isogenic study design	Possible, but generally slower and more resource-intensive
**Drug testing**	Useful for human-relevant mechanistic studies and screening workflows	Important for integrated pharmacology and in vivo efficacy, but may not predict human responses
**Disease modelling**	Particularly strong for human genetic disease and defined cellular phenotypes; increasingly useful for injury and remodelling models	Useful for some genetic and chronic diseases; strong for integrated injury, remodelling, and functional outcomes in vivo
**Cost**	Lower marginal cost per condition once established	Higher cost per experiment

Supplementary Table 2: hiPSC models of MI and IHD: model format, cell composition, injury method, and disease phase modelled

#	Paper title	Model format	Cell composition	Injury method	Disease phase(s) modelled	Ref
2D systems						
1	hiPSC-based platform for modelling cardiac ischemia	2D monolayer	hiPSC-CMs only	Hypoxia + metabolic stress followed by reoxygenation (low O_2, altered medium composition)	Acute I/R injury and CM death. Models mitochondrial dysfunction, Ca^{2+} handling changes, and CM viability	72
2	Modelling ischemia-reperfusion injury (IRI) in vitro using metabolically matured induced pluripotent stem cell-derived cardiomyocytes	2D monolayer with metabolic maturation	hiPSC-CMs only; matured in galactose- and fatty acid-containing medium	Ischaemia: 0% O_2 for 2h in glucose-free medium at pH 6.2 or 7.4. Reperfusion: 20% O_2 with glucose for 4h	Acute I/R injury and CM death. Provides well-defined CM death and mitochondrial injury endpoints	73
3	In vitro model of ischemic heart failure using human induced pluripotent stem cell-derived cardiomyocytes	2D monolayer with chemical ischaemia	Primarily hiPSC-CMs; some variants include CFs or ECs	Chemical ischaemia using metabolic inhibitors, with induction of CM arrhythmic phenotypes	Acute ischaemic-like metabolic stress and CM death. Focuses on ATP depletion, ROS and CM viability; not designed to follow later repair or remodelling	74
3D systems						
4	Bioreactor-based 3D human myocardial ischemia/reperfusion in vitro model: a novel tool to unveil key paracrine factors upon acute myocardial infarction	3D aggregates in a bioreactor	hiPSC-CMs	Controlled perfusion hypoxia (<0.4% O_2) with low glucose/nutrient-limited medium for 5h, followed by normoxic reperfusion	Acute I/R injury and early CM responses, including apoptosis, necrosis, sarcomere disorganisation, and mitochondrial damage	82
5	In vitro aged, hiPSC-origin engineered heart tissue models with age-dependent functional deterioration to study myocardial infarction	3D in hydrogel	hiPSC-CMs; young versus aged by culture duration	Oxidative H_2O_2 insult or 1% O_2 for 48h, followed by reoxygenation	Acute I/R-like injury with age-dependent susceptibility, ECM stiffening, better recovery in younger tissues, and reduced survival in aged tissues	83
6	Modelling acute myocardial infarction and cardiac fibrosis using human induced pluripotent stem cell-derived multi-cellular heart organoids	3D cardiac organoid	hiPSC-derived organoids containing CMs, CF-like stromal cells, ECs, SMC-/ epicardial-like cells	$CoCl_2$ and glucose depletion followed by 72h high glucose, high Ca^{2+}; fibrosis progression induced with TGF-β1	Acute MI-like injury and high glucose reperfusion, followed by organoid remodelling, CM death, inflammatory/ fibrotic transcriptional responses in fibroblast-like cells, ECM deposition, and neovascular changes	90

7	Human Tissue-Engineered Model of Myocardial Ischemia-Reperfusion Injury	Engineered heart tissue	hiPSC-CMs in collagen I and fibrinogen hydrogel	Nutrient deprivation and 0% O_2 for 6h, followed by normoxia, high glucose and Ca^{2+} for 3h	Acute I/R injury and high glucose reperfusion, with CM disarray and ROS accumulation	81
8	Biofabrication of advanced in vitro 3D models to study ischaemic and doxorubicin-induced myocardial damage	3D cardiac spheroids	hiPSC-CMs, primary human CFs and primary human coronary artery endothelial cells	0% O_2 for 20h followed by reperfusion at 5% O_2 for 17h	Acute I/R injury with CM death, reduced contractility and early MI-associated gene expression changes	86
9	Cardioids reveal self-organizing principles of human cardiogenesis	3D cardiac organoid (cardioid)	hiPSC-derived cardioids; self-organising “chamber-like” organoids containing CMs, ECs, CF-like cells	Focal cryoinjury applied to the cardioid	Localised necrosis and early healing/ remodelling. Captures acute CM death in a defined region followed by ECM deposition, fibronectin/collagen accumulation and partial functional recovery, mimicking aspects of infarction and early wound healing	65
10	Human cardiac organoids for the modelling of myocardial infarction and drug cardiotoxicity	3D cardiac spheroids	hiPSC-derived cardiac organoids containing CMs, ECs, and fibroblasts/stromal cells; self-organising dense structure (~150 µm radius)	Spontaneous hypoxic core due to O_2 diffusion limits in dense organoids cultured at 10% O_2 with 1 µM norepinephrine stimulation for 10 days to mimic infarct-like stress	Acute hypoxic injury and metabolic stress response. Captures a diffusion-limited hypoxic core (mimicking subendocardial ischaemia), ROS accumulation, cell death, and hypertrophy	87
11	Engineered model of heart tissue repair for exploring fibrotic processes and therapeutic interventions	Engineered heart tissue	hiPSC-CMs and -CFs in hydrogel	Cryoinjury	Acute damage followed by fibroblast migration, wound healing responses, and fibrotic remodelling	84
Bioengineered devices						
12	Endothelial extracellular vesicles contain protective proteins and rescue ischemia-reperfusion injury in a human heart-on-chip	Laminar cardiac tissues on film cantilevers	hiPSC-CMs	1% O_2 hypoxia with glucose deprivation for 3h, followed by 1.5h normoxia with glucose for reperfusion	Acute I/R injury and CM injury/death, followed by assessment of cardioprotective EC-derived extracellular vesicles	91
13	Biowire Model of Interstitial and Focal Cardiac Fibrosis	Aligned cardiac tissues on conductive wires	hiPSC-CMs and primary ventricular CFs	High CF:CM ratio to model interstitial and focal fibrosis; no hypoxia or I/R injury	Late-stage fibrotic remodelling with increased tissue stiffness, impaired contractility and calcium handling, and pro-arrhythmic features	94

14	Human cardiac fibrosis-on-a-chip model recapitulates disease hallmarks and can serve as a platform for drug testing	3D cardiac tissues anchored to flexible PDMS rods	hiPSC-CMs and primary human CFs	TGF-β1-induced fibrosis; no hypoxia or I/R injury	Fibrotic remodelling with reduced contractile function, collagen deposition and increased tissue stiffness, followed by anti-fibrotic drug testing	93
Heart-on-chip systems						
15	Human Heart Anoxia and Reperfusion Tissue (HEART) model for the rapid study of exosome bound miRNA expression as biomarkers for myocardial infarction	3D cardiac hydrogel with EC-lined vascular channel	hiPSC-CMs in human heart ECM hydrogel and hiPSC-ECs in vascular channel	Flow cessation and 0.1% O_2 for 3h, followed by restoration of normoxia and perfusion for 3h	Models acute MI-like flow restriction and reperfusion, with severe hypoxia during flow cessation followed by restored oxygenation/perfusion; captures early CM death and MI-associated gene expression changes	96
16	Cardiac ischemia on-a-chip to investigate cellular and molecular response of myocardial tissue under hypoxia	Aligned 3D cardiac tissues in a microfluidic chip with embedded microposts	hiPSC-CMs and primary human CFs	1% O_2 hypoxia for 24h, followed by reoxygenation for 24h	Acute I/R-like injury with CM loss; captures transcriptomic changes, altered contractility, and fibrotic response	97
17	Immune-integrated cardiac fibrosis-on-a-chip: a 3D microfluidic device for region-specific immune-cardiac crosstalk in the fibrotic heart	Region-patterned 3D cardiac tissue with valve-controlled cell delivery	hiPSC-CMs and -CFs; hiPSC-macrophages added region-specifically after patterning	No hypoxia or I/R injury; TGF-β treatment and spatial patterning creates scar, border-zone, and healthy-like regions	Post-MI fibrotic remodelling and immune–fibrosis crosstalk, with region-specific macrophage effects; does not model ischaemia or acute CM death	98
